# Fiber‐Optic Theranostics (FOT): Interstitial Fiber‐Optic Needles for Cancer Sensing and Therapy

**DOI:** 10.1002/advs.202200456

**Published:** 2022-03-23

**Authors:** Yang Ran, Zhiyuan Xu, Minfeng Chen, Wei Wang, Yang Wu, Jiexuan Cai, Junqiu Long, Zhe‐Sheng Chen, Dongmei Zhang, Bai‐Ou Guan

**Affiliations:** ^1^ Guangdong Provincial Key Laboratory of Optical Fiber Sensing and Communications Institute of Photonics Technology Jinan University Guangzhou 510632 China; ^2^ Department of Laboratory Medicine Guangdong Second Provincial General Hospital Guangzhou 510317 China; ^3^ College of Pharmacy Jinan University Guangzhou 510632 China; ^4^ Guangdong Province Key Laboratory of Pharmacodynamic Constituents of Traditional Chinese Medicine and New Drugs Research Jinan University Guangzhou 510632 China; ^5^ Department of Pharmaceutical Sciences College of Pharmacy and Health Sciences St. John's University Queens NY 11439 USA

**Keywords:** fiber optics, fluorescent sensing, hypoxia, photothermal therapy, precise tumor theranostics

## Abstract

Photonics has spurred a myriad of diagnostic and therapeutic applications for defeating cancer owing to its superiority in spatiotemporal maneuverability and minimal harm. The limits of light penetration depth and elusiveness of photosensitizer utilization, however, impede the implementation of the photodiagnostic and ‐therapy for determining and annihilating the deep‐situated tumor. Herein, a promising strategy that harnesses functional optical fibers is developed and demonstrated to realize an in vivo endoscopic cancer sensing and therapy ensemble. Tumor detection is investigated using hypoxia‐sensitive fluorescent fibers to realize fast and accurate tumor recognition and diagnosis. The tumor treatment is further performed by exploiting the endogenous photothermal effect of rare‐earth‐doped optical fibers. The eradication of orthotopic and subcutaneous xenografts significantly validates the availability of tumoricidal fibers. The strategy opens horizons to inspire the design of optical fiber‐mediated “plug and play” precise tumor theranostics with high safety, which may intrigue broader fields, such as fiber optics, materials, chemistry, medicine, and clinics.

## Graphical abstract

The limit of light penetration depth impedes the implementation of the photodiagnostic and ‐therapy. This work allows optical fibers to perform a “plug and play” detector and killer for determining and annihilating the deep situated tumor. The “freighters to fighters” twist opens a starting point for building the scalable platform dubbed as “medicine‐on‐a‐fiber.”

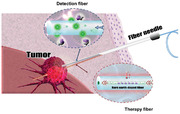

## 1 Introduction

Defeating cancer, which is one of the long‐sought‐after goals hitherto, necessitates rapid and precise oncodiagnosis and therapy, and summons innumerable pearls of wisdom from multiple disciplines to tackle various issues in the journey. Light, which has been a powerful tool for the treatment of diseases since antiquity,^[^ ^1^ ^]^ has illuminated a new era in malignant tumor imaging, sensing, and treatment because of the revolutionary advances in photonics and optics technology, which provides abundant degrees of freedom for light‐based tumor theranostics.^[^ ^2^, ^3^, ^4^, ^5^, ^6^, ^7^ ^]^ For example, optical spectroscopy can herald the presence and accurate position of the tumor aided by the recognition of tumor‐specific biomarkers^[^ ^8^, ^9^, ^10^, ^11^ ^]^ or the identification of the “fingerprints” belonging to the tumor‐associated antigens.^[^ ^12^, ^13^, ^14^ ^]^ The photon‐stimulated response is also an effective pathway for tumor‐orientated treatments, such as photothermal therapy (PTT) and photodynamic therapy (PDT).^[^ ^15^, ^16^, ^17^ ^]^ Light has created a superior platform for cancer theranostics by conferring remarkable advantages, such as high spatiotemporal controllability, ionization‐free, minuscule invasion, minor side effects, high versatility, and mitigation of cost and mental burden.^[^ ^3^ ^]^

However, a paramount challenge for photon‐based theranostics lies in the severe absorption and scattering of light by the human tissue. The low tissue penetration efficiency of light not only compromises the regulation of the laser illumination dose but also significantly impedes the application of the strategy for coping with large or deep‐situated tumor. In addition, despite the encouraging advancements in the photoreporting and photosensitizing drugs enabled by the prosperous development of nanotechnology, the application of nanomaterials in vivo still raises concerns about the drug uptake efficacy and safety, generally due to the vague understanding of the interplay between nanoparticles and organs, circulating systems, and tumors.^[^ ^2^, ^15^, ^18^ ^]^

The lack of light energy delivery associated with the tissue penetration depth can be wisely circumvented using a flexible interstitial light guide. Optical fibers, which constitute the intercontinental backbone telecommunication networks, can ship photons end‐to‐end with a negligible loss rate, providing excellent opportunities for internal tumor‐targeted light irradiation and harvesting. These features, such as electrical isolation, resistance to corrosion, hair‐like footprints, pliability, and versatility, further enable the optical fiber to match the niche for in vivo tumor determination and treatment that requires higher biosafety.^[^ ^19^ ^]^ As a result, optical fiber‐assisted PDT^[^ ^20^, ^21^ ^]^ and laser ablative hyperthermia^[^ ^22^ ^]^ are excellent paradigms for fiber tumor therapeutic strategies. More importantly, the optical fiber can be upgraded beyond a pure light guider when performing as the carrier and capsule for shuttling the photoreporters and photosensitizers without any hangover in the body. This strategy of new generation can provide a paradigm shift in the role of optical fiber from the “freighters” to “fighters” for precisely striking tumors yet leaving the normal tissue and organs in peace.

**Figure 1:**
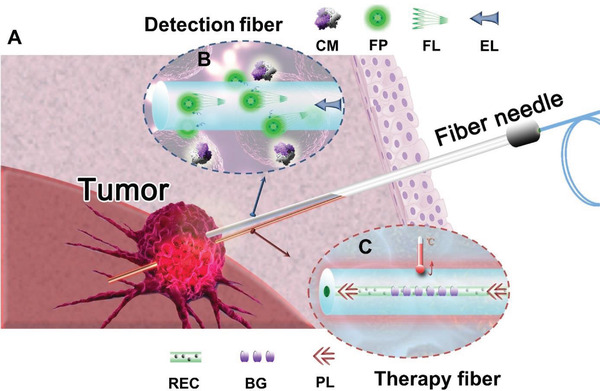
Diagram of optical fiber endoscopic needle for tumor sensing and therapy. A) The minimal invasive tumor theranostic needle containing the functional optical fibers for the navigation to a tumor. B) The in situ fiber tumor detector incorporating the utilities of excitation laser delivery, tumor‐specific sensing capability, and fluorescence acquisition. The cancer marker‐sensitive fluorescent probes were immobilized on the fiber surface. The functionalized fluorescent fiber can voyage to the designated position for identifying tumors through the interaction with the hypoxia markers. The excitation laser can be transmitted, and the tumor‐associated fluorescent signal can be gathered via the same fiber. The presence and location of the tumor can therefore be accurately disclosed. CM: cancer marker; FP: fluorescent probe; FL: fluorescent light; EL: excitation light. C) The photothermal therapeutic fiber vessel that seals the rare‐earth based photosensitizer within the core. The rare‐earth dopants can fully utilize pump laser and convert it into heat, enabling the direct hyperthermia attack on the tumor with high precision, efficiency, steerability, and safety. The build‐in fiber Bragg grating facilitates the monitoring of the local temperature during photothermal treatment. REC: rare‐earth doped core; BG: Bragg grating; PL: pump laser.

Herein, we propose fiber‐optic interstitial needles that carry hypoxia‐sensitive fluorescent probes and encapsulate rare‐earth dopants for combating tumors in vivo using an ensemble of endoscopic cancer sensing and PTT. The functional optical fibers, which had compact diameters of several hundred microns, can be organized and disposed of side‐by‐side into a commercial syringe needle, in which the total diameter can be controlled to less than 1 mm, to aid interstitial navigation (**Figure** **1**A). The detection fiber carrying the tumor marker‐sensitive fluorescent probes can rapidly scout the nearby hypoxia markers within the tumor (Figure 1B). Rare‐earth‐doped fibers were employed as flexible containers of photothermal sensitizers for annihilating tumors directly (Figure 1C). The fiber with self‐photothermal conversion allowed the local confinement of heat to reduce the risk underlying the direct laser thermalization of the tumor, which presented different types and morphologies that hindered the efficacy of heat production.^[^ ^15^ ^]^ In contrast to several recently reported attempts using functional material coatings as photothermal sensitizers,^[^ ^23^ ^]^ the rare‐earth dopants encapsulated by the fiber vessel can not only make full use of the pump light to raise the efficiency of light‐heat conversion (the treating power can be lowered from tens of watts to sub‐watts) but also mitigate safety concerns about the coating durability in the treatment. Furthermore, sealed rare‐earth dopants guarantee the long‐term use and recycling of therapeutic fibers in terms of immunity to oxidization and photobleaching. In addition, the maturing fiber device and sensor technology further potentiate the utility of the fiber theranostic platform. For example, the fiber Bragg grating (FBG) inscribed in the rare‐earth‐doped fiber can be tied to the PTT by its dedication to temperature monitoring. Considering the universality of the hypoxia and hyperthermia among solid tumors, our strategy provides a versatile and “plug and play” strategy for accurate theranostics of solid tumors, regardless of tumor types, origins, locations, and development stages, and needless to the additional injection of photosensitizer and reporter.

## 2 In Vivo Tumor Detection

**Figure 2:**
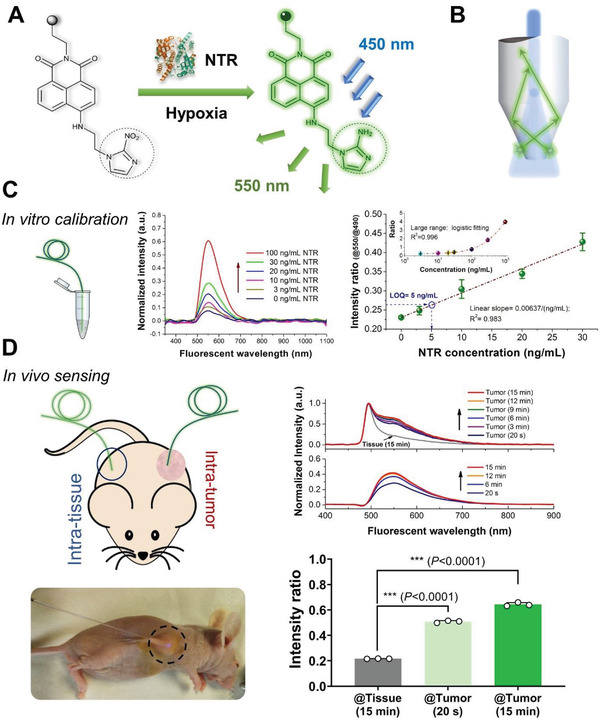
Fiber‐optic fluorescent sensor for in situ tumor detection. A) Principle chart of fluorescent probe for NTR sensing. NTR: nitroreductase. Under the hypoxia condition, which is often offered by the tumor microenvironment, the NTR probe can be reduced by the presence of NTR. Illuminated by the excitation laser of 450 nm, the reduced NTR probe emits fluorescent light with a peak wavelength of 550 nm. B) Schematic of the fiber tip design. A 600 µm diameter optical fiber was etched by one end to form a cone‐like structure. The tip diameter is ≈150 µm. The structure facilitates the transmission of excitation laser to the NTR probes in the vicinity of the cone region and either the collection of the fluorescent light from those probes. C) Sensing performance and calibration of the optical fiber tip for detecting NTR in vitro. The 550 nm fluorescent signal increases with the increment of the NTR density. For a large detection range (0–1000 ng mL^−1^), the curve could be well‐fitted by the logistic function (*R* ^2^ = 0.996). For a smaller detection range (0–30 ng mL^−1^), the curve could be approximately fitted in a linear regression function, *I*  =  0.00637 × *C* + 0.23101 ( *R* ^2^ =  0.983);  (I: intensity; C: concentration of NTR). The limit of quantification (LOQ) of the sensor can be obtained to ≈5 ng mL^−1^, according to the equation of $X_{\mathrm{LOQ}}=f^{-1}\;\left(\bar{y_{\mathrm{blank}}}+10\times \sigma \right)$; (*f*: function;$\;\bar{y_{\mathrm{blank}}}$: mean value of the blank sample tests; *σ*: standard deviation). Error bars are obtained by three dependent measurements. D) In vivo tumor detection using the functionalized fiber fluorescent sensor. The fiber sensor was harnessed to intervene the normal tissue and PANC‐1 tumor in the mirror spots, respectively. The intratumor manipulation was shown in the real experiment image. Other than the normal tissue that brings about little effective signal, the tumor allows the fluorescent signal accumulation with respect to time. The curve originates from the normal tissue providing a baseline for the quantitative analysis on the tumor‐associated fluorescent regarding the detection time. Even 20 s test for tumors showed a great significance in comparison to normal tissue, manifesting the fast and real‐time determination capability. Statistical analysis is performed by a one‐way ANOVA followed by Tukey's post hoc test. ***P < 0.001. The measurement values are randomly selected from the monitoring data with regard to each phase. Repeatability of the test results are validated by using the same fiber sensor that was refreshed and a similar designed fiber sensor (Figure S7, Supporting Information).

Nitroreductase (NTR), which plays a critical role in the proliferation, invasion, metastasis, and angiogenesis of malignant tumors, can be used as a biomarker of endogenous hypoxia within solid tumors.^[^ ^24^ ^]^ We adopted the fluorescent switch method to detect NTR. 1,8‐naphthalimide fluorophore was used as the “alarm lamp” because of its good stability, strong fluorescence, and high fluorescent quantum yield that is derived from the strong planar naphthalene nucleus structure.^[^ ^25^ ^]^ 2‐Nitroimidazole with high electron affinity (dashed circle) was conjugated to one end of the 1,8‐naphthalimide fluorophore as a switch that responds to NTR activation. As the nitro of 2‐nitroimidazole (an electron‐withdrawing group) was reduced to amino (an electron donor) by NTR, the fluorophore emitted strong green fluorescence at 550 nm, which was excited by 450 nm blue light excitation (**Figure** **2**A and Figure S1 and S2, Supporting Information). The remaining end of the 1,8‐naphthalimide fluorophore with an amino group was used to link the silica fibers.

We engineered one end of the optical fiber to a cone‐like tip using hydrofluoric etching to realize the effective delivery of the downstream excitation light and upstream fluorescence simultaneously (Figure 2B and Figure S3, Supporting Information). The launched 450 blue light was scattered at the cone region and interplayed with the NTR‐activated fluorophore in the immediate vicinity of the fiber tip surface via the evanescent field. The 550 nm green emission derived from the fluorescent scout was harvested through the cone structure. The cone structure was optimally designed with a length of 8 mm and tip diameter of 150 µm, which was one‐fourth of the original fiber diameter of 600 µm (Figure S4, Supporting Information).

To characterize the fluorescent sensing performance of the fiber tip, we conducted in vitro calibration by inserting the fiber probe into tube samples mixed with 1 mg mL^−1^ NTR probe and different concentrations of NTR (Figure 2C). The normalized intensity of the fluorescent emission peak increased, corresponding to an increase in the NTR density. For a wide NTR concentration range, the measured points of the peak intensity ratio can be well depicted by logistic fitting (*R* ^2^ = 0.996), which correlates to the increasing number of fluorophores activated by the NTRs. At the range of lower concentrations ranging from 0 to 30 ng mL^−1^, an approximately linear correlation was deduced, presenting a limit of quantification (LOQ) of 5 ng mL^−1^.

To demonstrate the feasibility of the fiber‐tip probe for tumor detection, we conducted an in vivo experiment using tumor‐bearing mice (Figure 2D). The fluorophore probes were covalently immobilized on the fiber tip to realize the in situ probing of the NTR produced in the solid tumor (Figure S6, Supporting Information). The fiber tip was manipulated to penetrate into tumor with a depth of 5–8 mm regarding the morphology of tumor. The normal tissue was also tested using the same way at mirror positions of the mouse body for comparison. At the normal tissue position, we monitored the gathered fluorescence for 15 min; however, no obvious signal was observed within the entire duration. For tumor detection, the fluorescent signal became brighter over time, indicating the continuous reduction of the NTR probe inside the tumor. Under the same reaction time of 15 min, the tumor trial showed significantly positive results compared to the normal tissue. Regarding the sensing calibration curve, the underlying NTR concentration in the tumor microenvironment was speculated to be ≈50 ng mL^−1^. Furthermore, it was remarkable to observe that even a 20 s detection (determined by the experimental operation duration) can effectively reflect a positive result, promising a real‐time diagnosis of the solid tumor. The NTR fiber fluorescent probe opens a straightforward route for the diagnosis of tumors, which can be conventionally realized by biopsy. Furthermore, the fiber probe can also be used to establish a precise criterion for deep‐seated tumors, facilitating subsequent treatment.

## 3 Fiber‐Optic PTT

**Figure 3:**
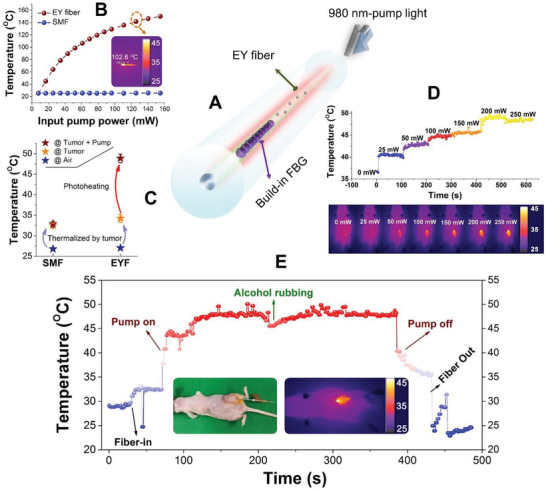
Characterization of the rare earth‐doped tumor PTT fiber. A) Diagram of the photoheating of rare earth‐doped fiber that was activated by the pump laser. A built‐in fiber Bragg grating monitored the temperature of the fiber heater. Yb: Ytterbium; Er: Erbium; FBG: fiber Bragg grating. B) Photon‐induced temperature changes in the air using the EYF and SMF as a function of the pump power. Inset: the IR record of the temperature of EYF under the pump power of 120 mW. C) The temperature changes logged by the built‐in FBGs regarding the scenario shift from air to the tumor and then in tumor photoheating. From air to tumor, both fibers were warmed up due to the tumor temperature. By contrast, as the pump was launched, only EYF elevated the temperature as a result of the pump‐heating conversion. D) Temperature increment dictated by the step changed pump power. Lower image: the IR images that reveal the thermalization with the increment of pump power. E) Sensorgram of the photoheating in a demo of treatment in vivo. The process includes fiber intervention, pump activation, tumor heating, and alcohol rubbing on the PANC‐1 tumor surface for rendering an instant cooling, pump shutoff, and fiber pulling out. The entire process was recorded in the Video S1 in the Supporting Information.

Rare‐earth‐doped optical fibers are widely employed in fiber amplifiers and lasers, in which photoheating mediated by the non‐radiative transition is customarily regarded as an adverse effect. Nevertheless, we inspected this effect from an opposite perspective, by selecting a kind of telecom‐compatible (with a diameter of 125 µm and a small numerical aperture) erbium and ytterbium codoped fiber with a higher light‐thermal conversion rate, to enable PTT fibers (Figures S8 and S9, Supporting Information). Meanwhile, the photosensitivity of the fiber can be exploited to inscribe the fiber Bragg grating, which serves as an integrated fast‐responsive thermomonitor and enables real‐time feedback potential (**Figure** **3**A and Figure S11, Supporting Information).

To verify the photoinduced heat strategy, we developed two types of optical fibers: a standard single‐mode fiber (SMF) and an Er/Yb codoped fiber (EYF). According to the measurements, the performance of EYF far outweighed the SMF by thermalizing itself to as high as 140 °C (Figure 3B). Infrared thermal imaging revealed hyperthermia of the EYF under 120 mW pumping in air. Next, we conducted an in vivo trial to understand the photoheating efficacy of these fibers in the tumor environment. Both fibers responded the tumor penetration‐induced temperature increment derived from the room temperature (26 °C) to the tumor temperature (around 35 °C). As a 200 mW pump went in, SMF presented a negligible response, whereas EYF suddenly rised its temperature to ≈50 °C. The higher thermal conductivity of the liquid internal environment of the tumor results in a lower self‐heating temperature, in contrast to the air condition. Nevertheless, it still satisfied the tumor necrosis temperature requirement (Figure 3C).

Then, we explored the characteristics of the EYF, which penetrated the tumor, by increasing the pump power from 0 to 250 mW. According to the sensorgram (Figure 3D), it can be observed that the self‐heating reached the up‐limit to ≈50 °C at 200 mW. The real‐time infrared thermal camera illustrates the temperature variation of the tumor with increasing power delivered to the active fiber end, firmly corroborating the temperature evolution. Infrared thermal images also qualitatively reveal the actuating range of the thermal therapy fiber, which could be well controlled within the tumor. Next, we performed real‐time monitoring of the entire therapy cycle (Figure 3E). As the fiber speared the tumor, the temperature abruptly increased, representing the fast response of the FBG sensor to the alteration of ambient temperature. We then activated the therapy fiber by injecting a 200 mW pump laser. Another abrupt step with a higher amplitude and sharper edge can be observed, indicating the instantaneous actuation of the photohyperthermia manifested by the rising edge spanning only several seconds. In the middle phase of therapy, we tried extremal alcohol rubbing on the tumor, which resulted in a transient cooling effect. The curve shows the corresponding cooling manipulation and reveals temperature restoration after alcohol volatilization. When the pump was turned off and the fiber needle was removed from the tumor, falling edges with a similar temperature amplitude were observed. Flash time photothermal actuation and removal facilitate the compression of the tumor therapy duration for risk and pain reduction in therapy. The temperature recovery ability provides robustness of the regime without being subjected to ambient interference.

**Figure 4:**
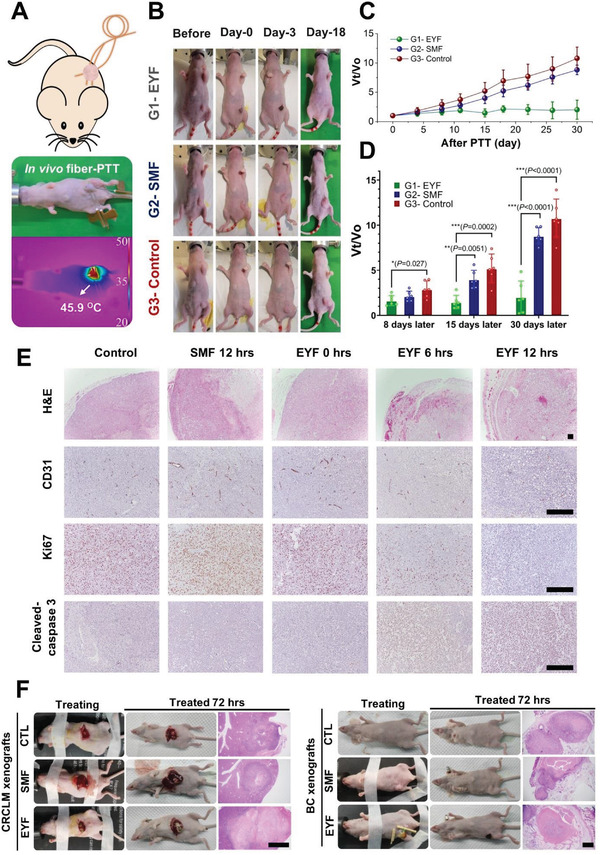
Anticancer efficacy of the rare‐earth‐doped fiber PTT. A) Principle chart of tumor treatment using self‐heated fiber. Three EYFs were utilized for generating a wide enough heating that covers the entire PANC‐1 tumor volume. Middle: The real treatment image. Bottom: IR image of temperature enhancement. B) Representative photographs of mice bearing PANC‐1 xenografts in different group sets before and after various treatments. Group 1: EYFs with 200 mW pumps; Group 2: SMFs with 200 mW pumps; Group 3: control without any treatment. C) Tumor growth inhibition curves (*V*_t_ indicates the volume at a measured time and *V*_0_ means the volume before treatment; the volume (mm^3^) = 1/2 × (tumor length) × (tumor width)^2^ of different groups of tumor‐bearing mice after treatments (*n* = 6). D) Statistical distinctions among the groups at several typical time ends. Statistical analysis is performed by one‐way ANOVA followed by Tukey's post hoc test. *P < 0.05; **P < 0.01; ***P < 0.001. E) Histology analysis of therapeutic effects in PANC‐1 tumor sections regarding the different treatments and periods (*n* = 6). Scale bar: 200 µm. F) Representative photographs of mice bearing HCT116 colon cancer liver metastases (CRCLM xenografts) and MDA‐MB‐231 breast cancer orthotopic xenografts (BC xenografts) in different group sets before and after various treatments, and H&E staining of tumor sections after various treatments (*n* = 6). Group 1: EYFs with 200 mW pumps; Group 2: SMFs with 200 mW pumps; Group 3: control without any treatment. Scale bar: 2 mm.

The efficacy of PTT using the fiber striker for tumor suppression and annihilation was further investigated. Nude mice bearing pancreatic cancer subcutaneous xenografts (20 g of average body weight and 100 mm^3^ of average pancreatic tumor volume) were randomly divided into three groups. We used three therapy fibers to spread the range of photoheating to cover the entire tumor (**Figure** **4**A). The three fibers were manipulated to penetrate the solid tumor‐bearing mice with the assistance of the intravenous needles. By pumping the three fibers simultaneously, the entire tumor was surrounded by heat mediated by the crossfiring coverage of the fibers (Figures S12–S14, Supporting Information). Untreated mice were used as negative controls. In addition to the EYF treatment using 200 mW pump power and the negative control groups, we set up an SMF group with the same treatment method as the EYF group to rule out the influence of the fiber intervention. We conducted a due course of treatment of the tumor at the beginning and third day, and each treatment course was maintained for 15 min. The tumor volumes and body weights of the mice were continuously monitored for every 3 d. The EYF‐treated mice exhibited a prominent burned black spot at the flank immediately after treatment and formed the eschar after 2 d (Figure 4B). Two weeks later, the eschar naturally went away, and the tumor vanished, leaving the healing tissue at the same spot (Figure 4B). By contrast, the tumor in the SMF group did not show any eschar and continued to grow in the days following the treatment. A similar trend was observed in the negative control group (Figure 4B). In addition, the EYF treatment group showed significant suppression of tumor growth compared to the other two groups. The SMF treatment group presented a slightly less remarkable slope than the control group in the tumor growth curve, which was probably ascribed to the penetration‐induced injury and disinfection (Figure 4C).

Quantitative analysis revealed the efficacy of EYF or SMF therapy at several typical time points (Figure 4D). On the eighth day, the EYF group starts to exhibit effective tumor suppression compared with the negative control group, although the eschars did not come off. On the 15th day, greater effects were obtained as the eschars shrank and came off. One month later, the two mice treated with EYFs were thoroughly cured. The other four mice showed effective restraint of tumor growth. By contrast, there was no significant difference between the SMF and negative control group throughout the treatment period. It is evident that the aforementioned penetration‐induced injury and disinfection could not thwart solid tumors alone.

Histological reports have further validated the therapeutic effects of EYFs. Hematoxylin and eosin (H&E) staining showed that SMF treatment had negligible effects on the histopathology of tumor tissues, whereas EYF treatment within 6 h led to hemorrhage and necrosis in both the tumor core and periphery, and these effects were further enhanced after 12 h of treatment. Immunohistochemical assays revealed that tumors treated with EYFs for 6 and 12 h drastically reduced the density of CD31^+^ microvessels and the number of Ki67^+^ proliferative tumor cells as well as cleaved Caspase‐3^+^ apoptotic tumor cells (Figure 4E). To further investigate the extended application of EYFs in tumor treatment, colorectal cancer liver metastasis exnografts (HCT116) and triple‐negative breast cancer orthotopic xenografts (MDA‐MB‐231) were constructed. Compared to the negative control and SMF groups, EYFs treatment led to a prominent burned black spot at the tumor loci immediately after treatment and forms the eschar after 2 d (Figure 4F). Consistently, H&E staining revealed that EYFs treatment resulted in extent tumor necrosis compared to the negative control and SMF groups, which indicated that the EYF therapy can effectively give rise to tumor necrosis and regression. More importantly, there were negligible changes in the body weight of tumor‐bearing mice in each treatment group throughout the treatment period (Figure S15, Supporting Information) and the cured mice without recurrence have survived for over 320 d (Figure S16, Supporting Information), indicating that the EYF and SMF therapies have a high safety profile. Tailoring of the fiber end shape could reduce the risk of the laser radiation due to the output of residual pump light (Figure S17, Supporting Information).

## 4 Conclusion

In summary, this work elucidates an integrated solution for direct cancer sensing and therapy. We employed the functional fibers, including hypoxia‐associated fluorescent fibers and rare‐earth‐mediated photothermal fibers, to precisely scout and attack tumors free of additional injection of photoreporters and photosensitizers. Positive tumor detection and regression results revealed the efficacy and safety of the proposed fiber theranostic concept, which runs in a “plug and play” paradigm (Figure S18 and Video S2, Supporting Information). The cure rate could be enhanced by assessing the status of tumor after treatment with aid of imaging system and conducting specific multiple courses of treatment to clear residuals. Moreover, importing the fiber needles outside‐in to the deep‐seated tumor in‐situ requires further investigations from the clinical and technical aspects. The detection and therapy efficacy using the fiber needles should be further evaluated regarding the atypia and heterogeneity of tumors. Nevertheless, this “wagons to weapons” twist can elevate the optical fiber beyond its current niche in the framework of cancer diagnosis and therapy by minimizing the body invasion and tissue damage compared with the traditional treatment of surgery. This study was also a starting point for building the scalable platform dubbed as “medicine‐on‐a‐fiber,” which may usher in more advanced strategies involving proven and cutting‐edge techniques of optoelectronics, materials and medicine. For example, fiber microfluidic devices will offer good opportunities for the exploration of fiber‐based medicine delivery^[^ ^26^, ^27^, ^28^ ^]^ and fibers with self‐photoactuation may facilitate navigation and manipulation of theranostic fiber needles in the body.^[^ ^29^ ^]^

## 5 Experimental Section

### Cell Lines and Cell Culture

Human pancreatic carcinoma cell line PANC‐1, human triple‐negative breast cancer cell line MDA‐MB‐231, and human colon cancer cell line HCT116 were obtained from American Type Culture Collection and cultured in Dulbecco's modified Eagle medium (Gibco, Grand Island) with 10% fetal bovine serum (ExCell Bio, Shanghai, China) and 1% penicillin‐streptomycin (Gibco) at 37 °C in a humidified atmosphere containing 5% CO2. The cells used in this study were authenticated as having no crosscontamination of other human cell lines using the Short Tandem Repeat (STR) Multi‐Amplification Kit (Microreader 21 ID System) and were tested negative for mycoplasma using the Mycoplasma Detection Set (M&C Gene Technology, Beijing, China).

### Animals

Four to six‐week‐old BALB/c‐Nu mice [BALB/cJGpt‐Foxn1nu/Gpt] were obtained from GemPharmatech (Nanjing, China). Animal experiments were approved by the Institute of Experimental Animal Ethics Committee of Jinan University (Approval number: 20200907‐010) and all mice were maintained in a specific pathogen‐free facility.

### Establishment of Pancreatic Carcinoma Xenografts

Pancreatic carcinoma xenografts were established according to a previously described procedure.^[^ ^30^ ^]^ Briefly, the human pancreatic carcinoma cell line PANC‐1 (5 × 10^5^ cells) suspended in 200 µL phosphate‐buffer saline (PBS, Servicebio, Wuhan, China) was subcutaneously inoculated in the flank of male BALB/c‐Nu mice. When tumors grew to ≈100 mm^3^, tumor‐bearing mice were randomly divided into three groups, including negative control group without any treatment, a control group with SMF treatment and therapy group with EYF treatment (*n* = 6). At the end of the experiment, the tumors were removed, weighed, photographed, and subjected to hematoxylin and eosin (H&E) staining and immunohistochemical analysis.

### Establishment of Orthotopic Breast Cancer Xenografts

MDA‐MB‐231 cells were harvested by trypsinization, washed twice with PBS and counted. Cells (5 × 10^5^ cells) suspended in 200 µL Matrigel (Corning, NY, USA) were injected into the intramammary gland fat pad of female BALB/c‐Nu mice. When tumors grew to ≈100 mm^3^, tumor‐bearing mice were randomized into three groups, including negative control group without any treatment, control group with SMF treatment and therapy group with EYF treatment (*n* = 6). At the end of the experiment, the tumors were removed, weighed, photographed and subjected to H&E staining.

### Establishment of Colorectal Cancer Liver Metastasis Xenografts

Colorectal cancer liver metastasis mouse models were established as previously described.^[^ ^31^ ^]^ HCT116 cells (1 × 10^5^ cells) suspended in 20 µL of Matrigel were injected into the left main lobe of the liver of male BALB/c‐Nu mice. When tumors grew to ≈100 mm^3^, tumor‐bearing mice were randomized into three groups: negative control group without any treatment, a control group with SMF treatment and a therapy group with EYF treatment (*n* = 6). At the end of the experiment, the tumors were removed, weighed, photographed, and subjected to H&E staining.

### Histological and Immunohistochemical Analyses

The fixed tumor tissues were embedded in paraffin and sectioned at a thickness of 5 µm. Thereafter, H&E staining was performed according to the standard procedures. For immunohistochemical analysis, sections were deparaffinized and subjected to antigen retrieval using ethylene diamine tetraacetic acid (EDTA) antigen retrieval solution (Beyotime). The slides were then blocked with 5% bovine serum albumin (BSA) for 1 h and incubated with anti‐CD31 (cat. AF3628, 1:200 dilution, R&D Systems), anti‐Ki67 (cat. 9449, 1:200 dilution, Cell Signaling Technology), and anti‐Cleaved caspase 3 (cat. 9664, 1:200 dilution, Cell Signaling Technology) antibodies overnight at 4 °C. The slides were washed with PBS, incubated with horseradish peroxidase (HRP)‐conjugated secondary antibodies including HRP‐conjugated antimouse (Cat. 7076, 1:400, Cell Signaling Technology), antirabbit (Cat. 7074, 1:400, Cell Signaling Technology), and goat (cat. HAF019, 1:400 dilution, R&D Systems) and then stained with a diaminobenzidine (DAB) kit, followed by counterstaining with hematoxylin. Images were acquired using an Olympus BX 53 microscope and analyzed using Image‐Pro Plus 6.0 software.

### Synthetic Procedures of NTR Fluorescent Probes

The synthesis procedure for the NTR probes is shown in Figure S1 in the Supporting Information. NTR (product number: N9284‐1MG‐PW) from *Escherichia coli* ≥90% (sodium dodecylsulphate‐polyacrylamide gel electrophoresis (SDS‐PAGE)), recombinant, expressed in *E. coli*. All reagents and solvents were purchased from commercial sources without further purification.

### Fiber Tumor Sensor Fabrication

Silica fibers with a cladding diameter of 600 µm were purchased from Thorlabs Ltd. The fiber coating was cleaved with a length of 1.5 cm from the fiber end. Thereafter, the optical fiber end was immersed into hydrofluoric acid (Guangzhou Chemical Reagent Factory, China, 40%, AR) to a depth of 2 cm, including the 1.5 cm long bare fiber and 0.5 cm long coated fiber. The duration of hydrofluoric acid etching was ≈4.5 h. The bare and coated fibers exhibited different corrosion rates due to the isolation of the polymer coating between the silica and HF; therefore, the fiber would present a column‐cone‐column three‐stage structure after hydrofluoric acid (HF) etching. The diameter of the bare optical fiber (smaller column) was etched to ≈150 µm at the end of the duration. The fiber was rinsed several times with deionized water and soaked in sulfuric acid (Tianjin Fuyu Fine Chemical, China, AR) for 0.5 h to remove the HF residue. The deionized water rinsing was repeated. The etched optical fiber was immersed in absolute ethyl alcohol (Tianjin Fuyu Fine Chemical, China, AR or acetone) to remove the remaining coating layer. A fiber cleaver was used to cut the smaller column and tailor the fiber end as a cone, as shown in Figure 2B and Figure S4 in the Supporting Information.

### Fiber Tumor Sensor Calibration

Pure PBS buffer solution and the PBS and 20 ng mL^−1^ NTR probe mixtures were prepared, respectively. The NTR samples were added into the PBS‐NTR probe mixtures with the concentrations of 3, 10, 20, 30, 100, 300, and 1000 ng mL^−1^ NTR. The mixed samples were put into the warm water (37 °C) for 1 h bathing. The etched end of the optical fiber was immersed into the mixed samples. Several spatial points were sampled with regard to the dipping depth of the fiber end (0, 0.5, 1, and 1.5 cm in the centrifuge tubes) to portray the error bars. The fiber sensor head was linked by the excitation laser of 450 nm (customized by the Shenzhen Innova Optoelectron. Tech., China, 450 nm laser diode) and spectrometer (purchased from Ocean optic, QE pro, United States). The setup can be found in Figure S3 in the Supporting Information. For extracting the fluorescent signal, a 450 nm filter should be set before the receiver to rule out the excessive excitation light intensity. The excitation light intensity was kept unchanged and then the final spectra were recorded by four times to portray error bars. The spectrum recorded in the pure PBS solution was regarded as the baseline and PBS‐NTR probe mixture was utilized as the blank samples. Since the fiber head at different positions showed different ability in collecting the excitation light which was mostly filtered, yet residual remained owing to the spectrum broaden as a result of the accumulative power saturation. The broadening of the excitation spectrum would affect the accurate fluorescent signal collected. Thereafter, an intensity ratio between 550 and 490 nm (@550 nm/@490 nm) was exploited as the marking value in the calibration. Given the spectra of lower concentrations (below 100 ng mL^−1^) of NTR possessed a higher excitation background (490 nm) than the fluorescent signals (550 nm), the curves could be normalized and the @550 nm/@490 nm ratios could be obtained directly from the normalized curves. Then the fluorescent spectra corresponding to different NTR concentrations could be obtained by subtracting the pure PBS spectrum. The recorded values were fitted by the calibrating functions. Using the calibrating function (*f*) and the blank sample data‐($\bar{y_{\mathrm{blank}}}$±*σ*), the LOQ was calculated by the equation of$\;X_{\mathrm{LOQ}}=f^{-1}\left(\bar{y_{\mathrm{blank}}}+10\times \sigma \right)$.

### Fiber Tumor Sensor Functionalization and In Vivo Determination

The functionalization of fiber tumor sensor was achieved by immobilizing the NTR probes on the fiber surface, following the procedures: immersion of the optical fiber cone in the sulfuric acid solution for 1 h. After completion, the optical fiber was rinsed in deionized water for 5 min, washed, and dried with absolute ethyl alcohol. The dried optical fiber was soaked in aminopropyl triethoxysilane (APTES) solution for 1 h. The fiber was then washed with absolute ethanol, rinsed in deionized water for 5 min, and dried. The optical fiber was immersed in glutaraldehyde solution for 0.5 h, soaked in deionized water for 5 min, and dried. The optical fiber was immersed in the NTR fluorescent probe (NP) solution for 1 h and the probe was cleaned and dried to complete the adhesion of NPs on the optical fiber. A scanning electronic microscope (Apreo 2 SEM, Thermo Fisher Scientific, the Netherlands) and a microscopic Raman spectrometer (DXR2, Thermo Fisher Scientific, the Netherlands) were employed to characterize the functional fibers. In vivo cancer determination follows the procedures below: The modified fiber head was placed inside the syringe needle. The syringe needle was first inserted into the right flank (normal tissue) of the mouse and then retracted leaving the optical fiber probe inside. After testing for 15 min, the spectra were recorded more than three times and used as a blank. Subsequently, the syringe was pulled out and penetrated the mouse tumor at the other flank and tested for 15 min in the same manner, a group of data was recorded every 20 s (*M* > 3). The normalized spectral lines were obtained by normalizing the original spectrogram, and the fluorescence signals could be clearly distinguished. Statistical analysis was performed between the tumor data recorded at 20 and 900 s and normal tissue data at 900 s. Each experiment was repeated using a different fiber sensor in the same batch of fabrication and the recycled fiber sensor enabled by piranha solution rinsing and refunctionalization as described above is shown in Figure S7 in the Supporting Information.

### Fabrication of Therapy Fiber

A piece of EYF (1 cm in length and 125 µm in diameter) was unilaterally spliced with a telecommunication fiber (SMF, 125 µm in diameter) by a fiber splicer (Fujimura Co., Ltd., 21S, Japan). The SMF undertook the role of transporting the pump light and the monitor signal. The EYF was then inscribed with a 5 mm length Bragg grating using as the excimer laser grating writing system. The system comprised a 193 nm excimer laser (Compex Pro 110F from Coherent Inc. USA) and a phase mask (1072.15 nm pitch, Ibsen, Denmark). The fiber end was fused to a hemisphere by arc discharge using a fiber splicer. The standard arc discharge power was set with a 2 s duration.

### Therapy Method

*Fiber Therapy Setup (Figure* S9 *, Supporting Information)*: The conventional band (1530–1565 nm) amplified spontaneous emission source (Shenzhen Innova Optoelectron. Tech., China) with an average power of −25 dBm was launched into the optical link via a circulator (Thorlabs, Inc., USA; the optical path was unidirectional, 1‐2‐3). The amplified spontaneous emission (ASE) source light and the 980‐pump laser light (Shenzhen Innova Optoelectron. Tech., China) were transported parallel to the therapy fiber using a wavelength division multiplexer (Thorlabs, Inc, USA, 980/1550). The reflective spectrum of the built‐in FBG was interrogated using the optical spectrum analyzer (AQ6370D, from Yokogawa Co., Ltd., Japan). IR imaging during photothermal treatment was acquired by the photothermal imager (FOTRIC 225s, FOTRIC, China) to reference thermal actuation. Fiber therapy manipulation: isoflurane (RWD Life Science Co. Ltd. China) was guided to the tumor‐bearing mouse using a small‐animal anesthetic machine (R580S, RWD Life Science Co. Ltd. China). The flux was controlled at 2 L min^−1^ and the density of isoflurane was 1.5%. The therapy fiber was inserted into a 1 mm syringe to allow the penetration into the tumor. The proper penetration angle and the position the tumor size and morphology were selected to enable the range of action that could cover the entire tumor. A low power pump of 30 mW was launched to visualize the EYF because of the lateral green emission resulting from the upconversion of rare‐earth dopants. Assisted by green upconversion florescence, the relative position between the therapy fiber and the tumor could be adjusted. The auxiliary syringe was then withdrawn, leaving the therapy fiber inside the tumor. The pump was elevated to a power of 200 mW and the therapeutic phase was started. Finally, the therapy fiber was withdrawn from the tumor after the 15 min therapy duration. Therapeutic results were evaluated by continuously measuring tumor size and body weight every 3 d.

### Simulation of Fiber Thermal Actuation

The heating area mediated by the EYF was simulated using COMSOL. As the 980‐pump laser light was input into the PTT probe, a small part of the pump energy was converted into green upconversion fluorescence and the ASE emissions. The major remaining pump light energy is converted into heat, which leads to an increase in the fiber core temperature. The heat then quickly spreads out of the cladding and thermalizes the surrounding biological tissue (or tumor) through the heat conduction and a small amount of body fluid convection. The PTT can be realized by creating a necrosis area in the tumor.

The heat source model inside the PTT probe can be expressed as follows^[^ ^32^, ^33^ ^]^

(1) $$q\;\left(r\right)=\left\{\begin{array}{c} \eta \cdot \alpha \cdot P\left(r,\;z\right)\;\left(0<r\leq r_{1}\right)\\ \;0\;\left(r_{1}<r\leq r_{2}\right) \end{array}\right.$$

where *η* is the thermal conversion efficiency, *r*_1_ and *r*_2_ are the radii of the core and cladding respectively, *α* is the pump light absorption coefficient, and *P* (*r*, *z*) is the spatial distribution of the pump light in the active fiber which can be expressed as follows:

(2) $$P\left(r,\;z\right)=P_{0}\cdot P_{t}\left(r\right)\cdot exp\left(-\alpha \cdot z\right)$$

where *P*_0_ is the incident pump power and *P*_t_(*r*) is the normalized energy distribution of the core fundamental mode.

Assuming that the fusion loss between the active fiber and passive fibers is very small and the fiber is straight, the higher‐order‐mode pump‐light is negligible. In addition, the actual range of heat conduction is much larger than that of the core region, which acts as the heat source. Consequently, the model was simplified in simulation. The 1 cm long active fiber probe is saturated for pump absorption, and the axial temperature distribution of the probe is no longer considered. Thus, the model can be considered to a simple 2D axisymmetric model and the core region generates heat uniformly in the radial direction.

When the input power *P*_0_ was set at 200 mW, calibrated by the power meter, through a 1 cm active optical fiber, a near 70 mW was converted into thermal energy. Using a finite element analysis model, the temperature distribution was simulated along the radial direction of the photothermal probe penetrating the human liver, as shown in Figure S12 in the Supporting Information. The other relevant parameters are as follows: *k*_glass_ = 1.4 W m^−1^ K^−1^, *C*_pglass_ = 730 J kg^−1^ K^−1^, *ρ*_glass_ = 2210 kg m^−3^, *k*_liver_ = 0.52 W m^−1^ K^−1^, *C*_pliver_ = 3540 J kg^−1^ K^−1^, *ρ*_liver_ = 1079 kg m^−3^, *ρ* is the density of the material, *C*_p_ is the specific heat capacity at atmospheric pressure, and *k* is the thermal conductivity. The model was set with a radius of 5 cm as the boundary of the liver tissue and the probe heating area was located at the center. The initial temperature of all positions was set at 310.15 K (37 °C).

### Statistical Analysis

Separate experiments were conducted more than three times. Statistic is presented as mean ± standard error of the mean (SEM). All statistical data were processed using GraphPad Prism 7.0 software (GraphPad Software, La Jolla, USA). Differences among the three groups were evaluated using one‐way analysis of variance (ANOVA) followed by Tukey's post hoc test. *P < 0.05 was considered a significant difference.

## Conflict of Interest

The authors declare no conflict of interest.

## Supporting information

Supporting information

Supporting information

Supporting information

## Data Availability

The data that support the findings of this study are available from the corresponding author upon reasonable request.
