# Supplementary material for: Fiber‐Optic Theranostics (FOT): Interstitial Fiber‐Optic Needles for Cancer Sensing and Therapy
Source: Adv Sci (Weinh). 2022 Mar 23;9(15):2200456. doi: 10.1002/advs.202200456 (PMC9130922; doi:10.1002/advs.202200456)
Supplement: Supplementary file 1 — Supporting information [file ADVS-9-2200456-s002.pdf]

ADVANCED SCIENCE
Open Access

Supporting Information

for *Adv. Sci.*, DOI 10.1002/advs.202200456

Fiber-Optic Theranostics (FOT): Interstitial Fiber-Optic Needles for Cancer Sensing and Therapy

Yang Ran, Zhiyuan Xu, Minfeng Chen, Wei Wang, Yang Wu, Jiexuan Cai, Junqiu Long, Zhe-Sheng Chen, Dongmei Zhang and Bai-Ou Guan**

Supporting Informations for

Fiber Optic Theranostics (FOT): Interstitial fiber-optic needles for cancer sensing and therapy

Yang Ran, Zhiyuan Xu, Minfeng Chen, Wei Wang, Yang Wu, Jiexuan Cai, Junqiu Long, Zhe-Sheng Chen, Dongmei Zhang, Bai-Ou Guan**

Supplementary Text

Mechanism of the NTR probe in NTR sensing

Nitroreductase (NTRs) took reduced nicotinamide adenine dinucleotide phosphate (NAD(P)H) as electron donors via single-electron reduction reaction conversing electron-withdrawing nitro reduce to electron-donating hydroxylamine or amino selectively, which results in a significant electronic conjugation effect activating strong fluorescence effect [34-36] (Figure S2A). Here, 1,8-naphthalimide fluorophore was conjugated to an NTR activity-based group and amino for conjugating with other materials, e.g., silica surface of optical fiber, as shown in Figure S2B. Due to the high electron affinity, 2-Nitroimidazole was chosen to be the NTR activity-based group. 2-Nitroimidazole presents a unique response to NTRs under hypoxia, lying in the consolidation of the intermediate product and promotion to the further reduction steps [37-38]. As the nitro of 2-Nitroimidazole (an electron-withdrawing group) was reduced to amino (an electron donor) by NTR, the compound emits strong fluorescence peaking at 550 nm excited by 450 nm irradiation. Figure S2C presents the NTR activated fluorophores with different concentrations of 0, 10, 50, 100, 300, and 1,000 ng/mL (Left to Right) under 450 nm light excitation.

Design of the Sensing fiber

According to Figure S4A, the fluorescent spectrometer presented the fiber detection with the original diameter of 600 μm without the 450 filter. The 450 nm excitation light could be clearly observed that outweighed the activated fluorescent signal at 550 nm. The excitation light power was elevated to create the higher fluorescent signal, however, intensity saturation would be encountered if the signal intensity went up to 60,000 counts and thus broadened the excitation spectrum, which led to the submerging of the fluorescent signal. As a consequence,

we added a 450 nm filter in the setup to rule out the excitation light as much as possible. In the following, we portrayed the calibration curve of the 600 μm fiber with a flat end using the maximum incident power of 60,000 counts. It could be seen from Figure S4B that the fluorescent signal enhanced with the increase of the NTR density. And at higher density, the fluorescent signal could significantly surpass the hangover of the excitation signal. However, for a lower density range, a higher LOQ was obtained to 56 ng/mL. That is due to the cylindrical fiber can only support the excitation and collection of the NTR probe immobilized on the end-face, which has a limited area. To overcome this issue, the lateral surface of the optical fiber can be fully utilized by means of fiber tailoring. We used the hydrofluoric acid to etch the fiber to achieve the lateral excitation and collection via the evanescent field of the fiber. With the optimization of the end-face tailoring, as shown in Figure S4D and F, the LOQ of NTR could reach 5 ng/mL (Figure 2C), an order of magnitude lower than the fiber without etching (Figure S4c and S4e).

Ex-vivo sensing

A piece fresh lean pork was prepared as an *ex-vivo* tissue substrate. The PBS buffer was used to deliver the baseline. The NTR probe- PBS mix sample was taken as a blank (NTR=0). The NTR-NTR probe-PBS mixture samples were prepared with the NTR concentrations of is 10 ng/mL, 50 ng/mL, 300 ng/mL and 1,000 ng/mL. Those samples were injected into pork tissue with a syringe at different positions (Figure S5). The etched optical fibers was inserted into tissues and reached the inject spots. Measurements were conducted as described in the calibration section. Similar to the *in vitro* calibration, spectra regarding *ex-vivo* experiment showed that the position with denser NTR sample resulted in stronger fluorescent intensity. The statistical analysis is performed between the 10 ng/mL (50 ng/mL) and blank samples. Both 10 ng/mL (P=0.0137) and 50 ng/mL (P=0.0002) sample positions exhibited significantly positive results in contrast to the blank one, shedding light on the effective determination of NTR using the fiber tip probe in the tissue.

Characterization of the fiber functionalization with NTR probes

We conducted the immobilization of the fluorophore probes on the fiber tip to realize the *in-situ* probing of the NTR produced in solid tumor. The rest amino end of 1,8-naphthalimide fluorophore allowed the integration of the optical fiber NTR probe by adopting the frequently-used covalent bonding method. After fluorophore immobilization, the functional optical fiber could be characterized by the fluorescent microscope. In contrast to the bare fiber without functionalization (Figure S6A and B), the NTR probe-decorated fiber emitted brighter green fluorescent light (Figure S6C) under microscope as was activated. Non-covalent binding of

NTR probe could be mostly removed after DI washing, making the fiber even dimmer (Figure S6D). By contrast, the covalent bonding NTR probe survived after DI washing (Figure S6E), validating the robustness of the functional fiber probe. The immobilized fiber tip probe can not only allow the direct detection in the tumor but also, in principle, address the concerns about biosafety attributed to the dissociative NTR fluorophore probes via body injection.

Mechanism of energy level transition of Erbium and Ytterbium structure

As shown in the inset of Figure S8, both Yb^{3+} and Er^{3+} in the fiber can absorb 980 nm photons in their ${}^2F_{7/2}$ and ${}^4I_{15/2}$ levels and excite them to ${}^2F_{5/2}$ and ${}^4I_{11/2}$ levels, respectively [39-41]. The emission spectrum of the Yb^{3+} locates in 1060 nm band. The excited Er^{3+} quickly transits to the metastable level ${}^4I_{13/2}$ through a non-radiative process, which results in the generation of heat[42]. Then, the photon emission at 1550-band is mediated by the decay of Er^{3+} in metastable ${}^4I_{13/2}$ down to the ${}^4I_{15/2}$ [43]. In Er/Yb co-doping system, the higher density clusters of Yb^{3+} dedicate the efficient 980 nm pump absorption without being subjected to the aggregation-based quenching effect [44]. The Er^{3+} nested by the Yb clusters drew the energy from the upper energy level of Yb^{3+} through the ${}^4I_{11/2}$ and ${}^4F_{7/2}$ levels, yielding the enormous heat mediated by non-radiative transition from ${}^4I_{11/2}$ to ${}^4I_{13/2}$ and the upconversion emissions of 525 nm and 550 nm relying on the transitions from ${}^2H_{11/2}$ and ${}^4S_{3/2}$ to ${}^4I_{15/2}$, respectively. [45-51].

Characterization of the PTT fibers

The thin layer of the silica cladding performs an excellent thermal conductor that transfers the heat outside and maintains the heat equivalence between the inner and outer fiber. To verify the photo-induced heat strategy, we had systematically investigated three kinds of optical fibers, the Yb mono-doped fiber (YDF) which was in addition to the SMF and EYF described in the main text. In contrast with the SMF, both rare-earth doped fibers presented effective absorption to the pump power because of Yb dopants (Figure S9A). The slight distinction between the EYF and YDF lied in the Er ions that gave rise to the pump energy transfer. Then, we investigated the temperature sensitivity of the FBGs written in those kinds of fibers. The nearly identical temperature sensitivity around 11 pm/°C allows for the quantitative analysis of the pump-heat conversion rate using different fibers (Figure S9B). Leveraging the built-in FBGs as the thermometers, the fibers were pumped under the room-air condition. According to the measurements, the performance of EYF outweighs the other two counterparts far ahead because of large amount of heat generated by the non-radiative transition of Er^{3+} (Figure S9C). We have then conducted the *in vivo* trial to understand the photo-heating efficacy of those fibers in the tumor environment (Figure S10). All of the FBGs responded to the tumor penetration-induced temperature elevation (26 °C to 35 °C). As the

200 mW pump was launched, YDF and SMF presented tiny response whereas EYF sudden rised its temperature to approximately 50 °C. Higher thermal conductivity of the liquid endo-environment of the tumor derives the lower self-heating temperature in contrast to air condition.

Characterization of the action range of the PTT

The simulation results (Figure S12, one fiber) show that, the heat energy will accumulate with the increase of heating time. Compared with the shorter time heating (5 min), the longer time heating (30 min) leads to a higher temperature of the liver tissue and a larger effect region. We further illustrate the heating region by the isothermal lines. When the temperature of a tissue increases to 42 °C, irreversible tissue damage occurs and heating of tissues to a temperature of 42~46 °C for 10 min will result in the cell necrosis [52-53]. We set the 45 °C isothermal line as the approximate edge of the necrosis area. The simulation results (Figure S12, one fiber) show that as the probe is heated for 5 min, the radius of necrotic tissue area is only about 200 μm; when the probe is heated for 30 min, the radius of necrotic tissue area will be expanded to about 500 μm. This result agrees well with the real experimental result.

However, a necrotic tissue area with 500 μm radius is not enough to accomplish the effective therapy for clinical tumor treatment. Therefore, we further simulate the overlapping results (Figure S12, three fiber) of three fiber probes which is achieved in the real treatment process. Similarly, 45 °C is used as the damage threshold temperature to define the necrosis area. Compared with the necrotic range using one single probe, the necrosis area under the overlapping of the three probes at 5 min had no significant improvement, with a radius of about 300 μm. However, when the heating duration comes to 30 min, the effect range of each probe overlaps with each other, and the actual tissue volume is significantly increased by about 100 times (The radius is about 5 mm compared it with the area of a circle), which is eligible to cover the small size solid tumors.

To figure out the action range of the PTT fiber, hstological analysis of therapeutic effect area was presented in the Figure S13 regarding the PANC-1 tumor after PTT. The wound caused by the invasion of the syringe and optical fiber could be seen as red-dashed. The size of actual necrosis range was approximately 1 mm.

We set up two groups (three EYFs and one EYF) to conduct PTT for comparison. The control group was also included. According to Figure S14, the EYF*1 group exhibited a certain degree of tumor suppression in contrast to the control group because a portion of the tumor cells was killed. A statistical significance between the EYF*1 group and control group

could be observed at the 30th day after treatment (*P*<0.01), whereas the curve of EYF*1 group kept growing as time went by, denoting the proliferation of the untreated tumor cells due to the limited therapy area using just one PTT fiber. The EYF*3 group showed significant difference to the EYF*1 group at both 15th (*P*<0.01) and 30th day (*P*<0.001) after the PTT.

Evaluation of the biosafety from the concern of the residual pump

The residual pump laser output may arouse safety concerns if it is not well-treated, even though EYF can absorb a substantial majority of the pump power. Commercial fiber fused splicer allows us to tailor the morphology of the fiber end-face to a hemispherical structure, which scatters the light that was otherwise concentrated in the fiber core for reducing the dose density (Figure S17A). We evaluated the viability of the power decentralization by aligning the fiber end to the normal tissue of the mouse directly (Figure S17B). One active fiber aimed at the tissue from outside (right flank), and another active fiber intervened the tissue (left flank). A 300 mW pump laser, which is higher than the power used in this PTT, was launched for 1 h and no noticeable burning injury could be observed in both exposure spots after 24 h, indicating high biosafety for the fiber endoscopic treatment.

Supplementary figures:

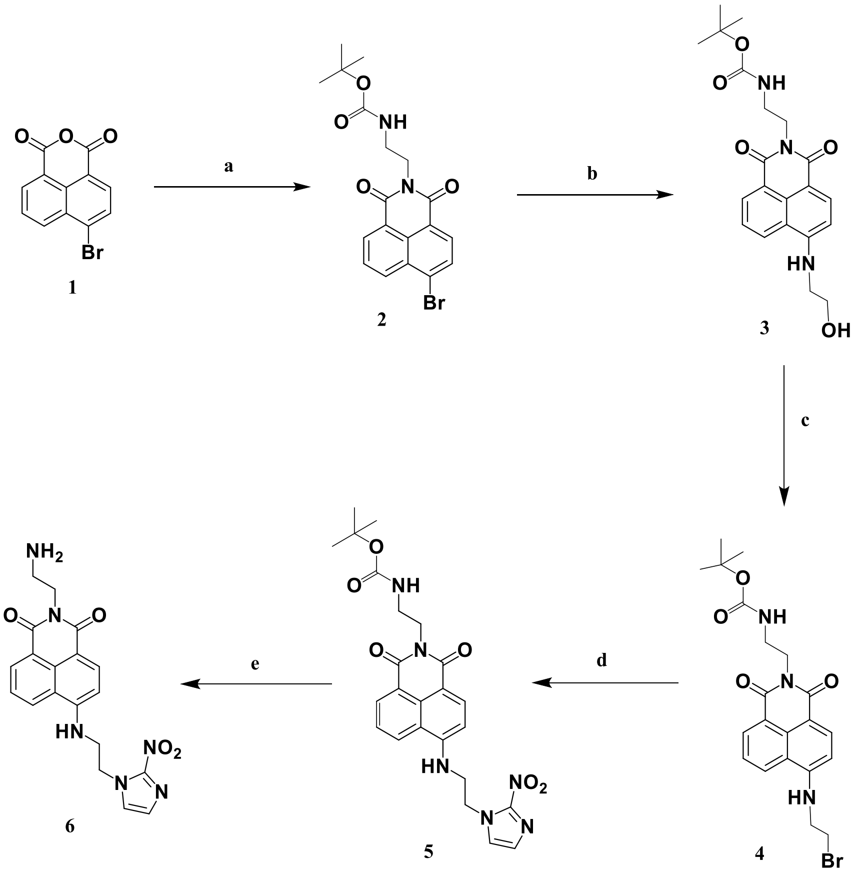

Figure S1. Synthetic procedures of NTR fluorescent probes. Compound-1: 4-bromo-1,8-naphthalic anhydride. Compound-2: Compound-2 was synthesized according to the published methods [54]. A mixture of 4-bromo-1,8-naphthalic anhydride and (2-aminoethyl) carbamic acid tert-butyl ester (1.5 eq) in EtOH was refluxed for 4 h under Ar atmosphere. The reaction product was then cooled down to the room temperature. Removing the solvent after the concentration of the mixture, the residue of coarse product was obtained and subsequently purified by the column chromatography on the silica gel till the white solid yielded. Compound-3: The compound-2 was dissolved in 2-methoxyethanol. Then, ethanolamine (2 eq), Cs_2CO_3 (1.2 eq), and KI (0.1 eq) was added into the compound-2 solution under Ar atmosphere. The mixture was stirred for 5 h under reflux. The reaction product was cooled to room temperature. Removing the solvent after the concentration of the mixture, the residue of coarse product was obtained and subsequently purified by the column chromatography on the silica gel till the orange solid yielded. Compound-4: A mixture of compound 3, Carbon tetrabromide (1.5 eq), and triphenylphosphine (1.5 eq) was stirred at 0 °C under argon. Then DMF was added and stirred at RT for 2. The reaction mixture was diluted with EA and washed by water and brine. The organic layer was dried by anhydrous sodium sulfate, and then filtered and concentrated for removing the solvent. The crude material was purified by flash chromatography till the yellow solid yielded. Compound-5: A mixture of compound 4, 2-Nitroimidazole (1.5 eq), KI (0.5 eq), and K_2CO_3 (2 eq) was stirred in DMF at 60 °C for 48 h. The mixture was then diluted by DCM and washed by water and brine. The organic layer was dried by anhydrous sodium sulfate, and then filtered and concentrated for removing the solvent. The crude material was purified by flash chromatography till the yellow solid yielded. Compound-6: A solution of compound 5 in TFA/DCM (1:1) was stirred at RT for 1 h. The

solution was neutralized by $NaHCO_3$ solution to a pH value between 7 and 8. The mixture was diluted by DCM and washed by brine. The organic layer was dried by anhydrous sodium sulfate, and then filtered and concentrated for removing the solvent. The crude material was purified by flash chromatography till the orange-yellow solid yielded.

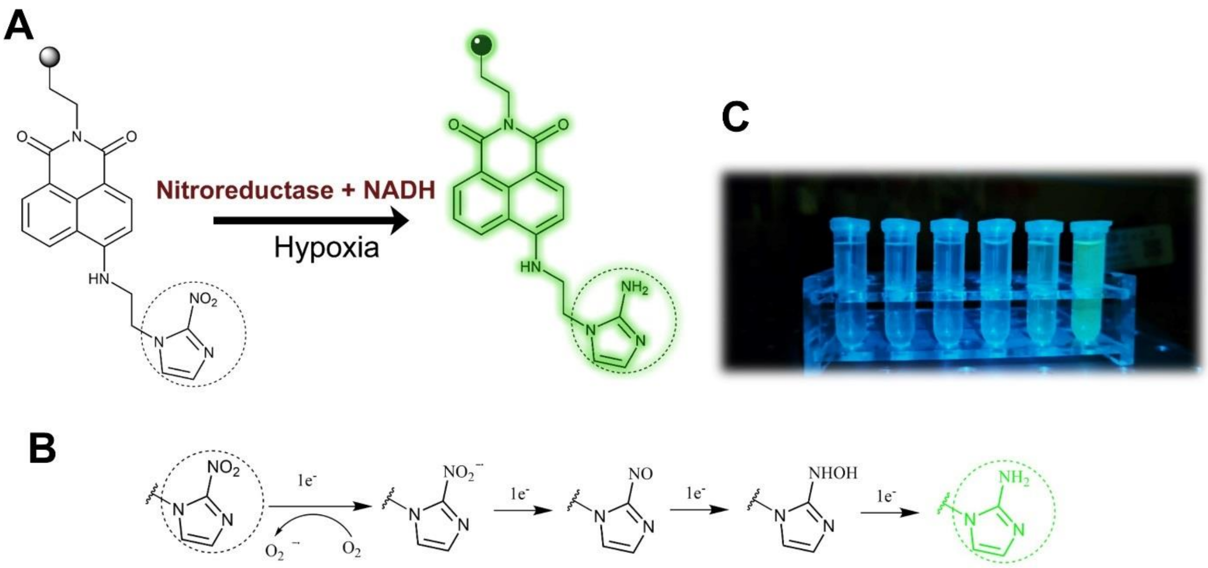

Figure S2. Mechanism and characteristics of NTR probes. A) Principle of NTR probe in detection NTR under the scenario of hypoxia and NADH. B) The electron transfer of the functional group in the fluorescent activation. C) NTR activated fluorophores with different concentrations of 0, 10, 50, 100, 300, and 1,000 ng/mL (Left to Right) under 450 nm light excitation.

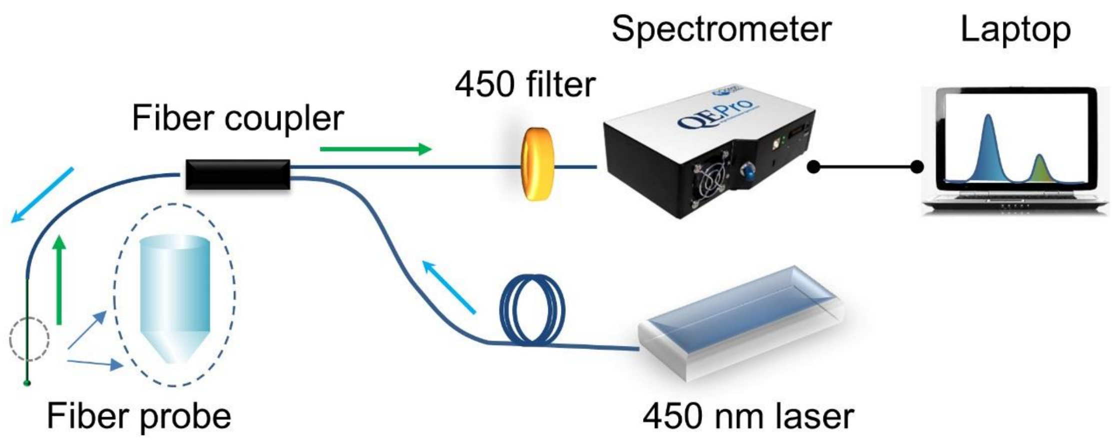

Figure S3. Diagram of experimental setup for fiber tumor sensing. The 450 nm laser is delivered to the fiber probe end through the fiber coupler and the 550 nm fluorescence is collected by the other port of the fiber coupler. With the assistance of a 450 nm filter, the fluorescence signal with a lower signal background enters the fluorescent spectrometer and the data are logged by the laptop connected.

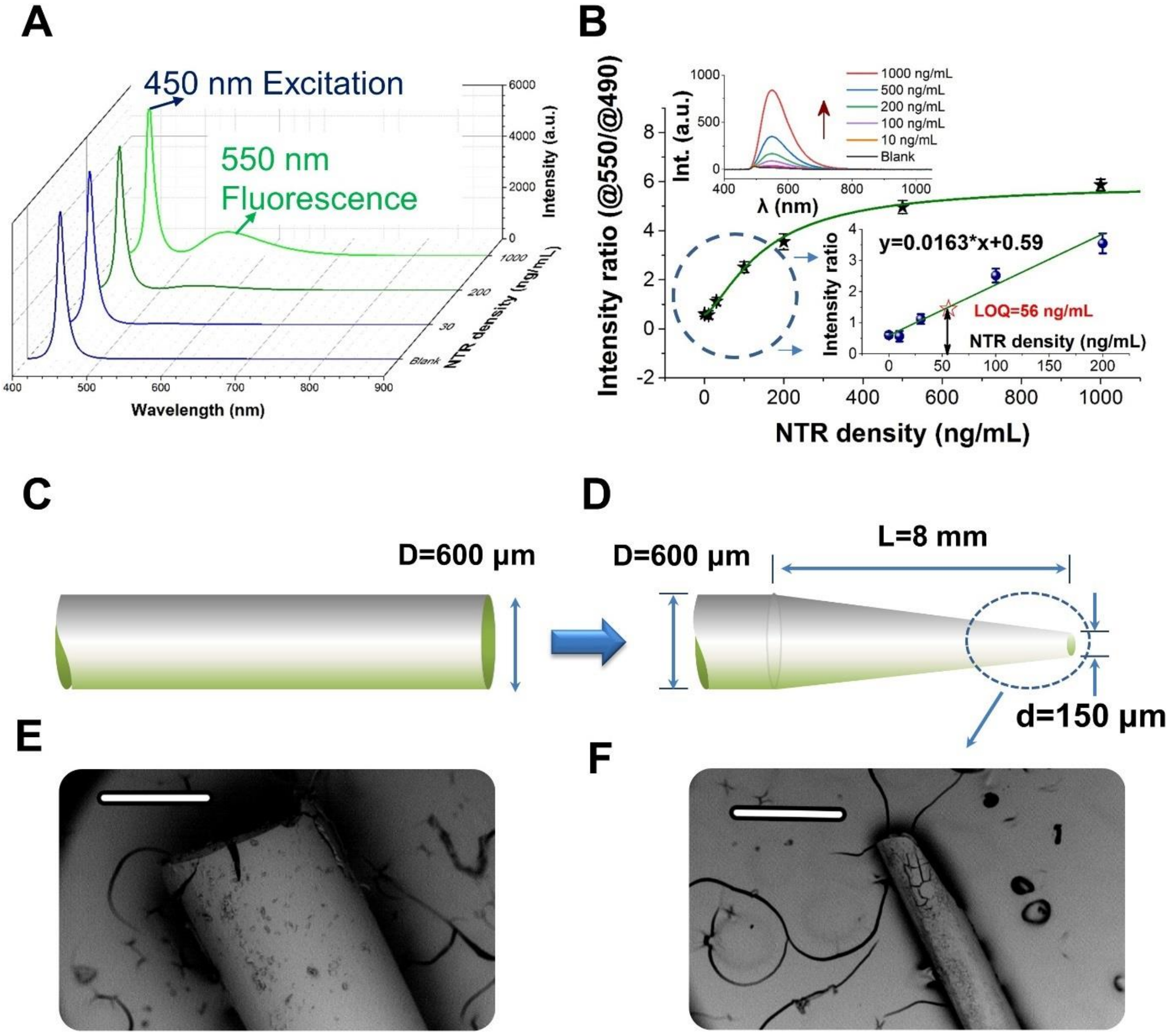

Figure S4. Characterization of the fiber and setup. A) The collected spectra regarding the different NTR density using the unetched fiber without 450 nm filter. The 450 nm excitation signal would overwhelm the fluorescent signal as the excitation power goes to the saturation intensity of 60,000 counts. B) The calibration curve obtained using the unetched fiber with the 450 nm filter. At lower density range, the LOQ to NTR is about 56 ng/mL. C) Diagram of the unetched fiber with the cladding diameter of 600 μm. D) Diagram of the ethced fiber with a cone like structure. The length of the cone is 8 mm and the tip diameter is 150 μm. E) SEM image of the unetched fiber. F. SEM image of the fiber cone. Scale bars indicates a length of 500 μm.

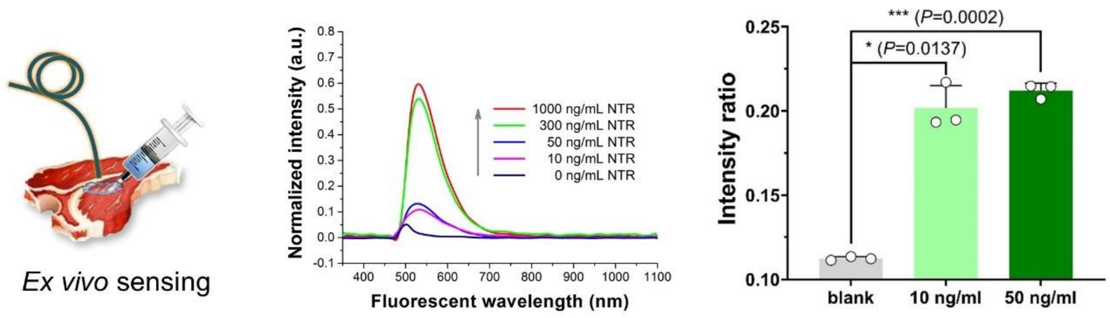

Figure S5. *Ex vivo* sensing. The NTR and NTR probes mix is injected into the meat tissue after the reaction. And then the fiber is interstitially inserted into the injection spot. The fluorescent signal goes up as the increament of the NTR density. Both 10 ng/mL (*P*=0.0137)

and 50 ng/mL (P=0.0137) samples exhibit significantly positive results in contrast with the blank sample.

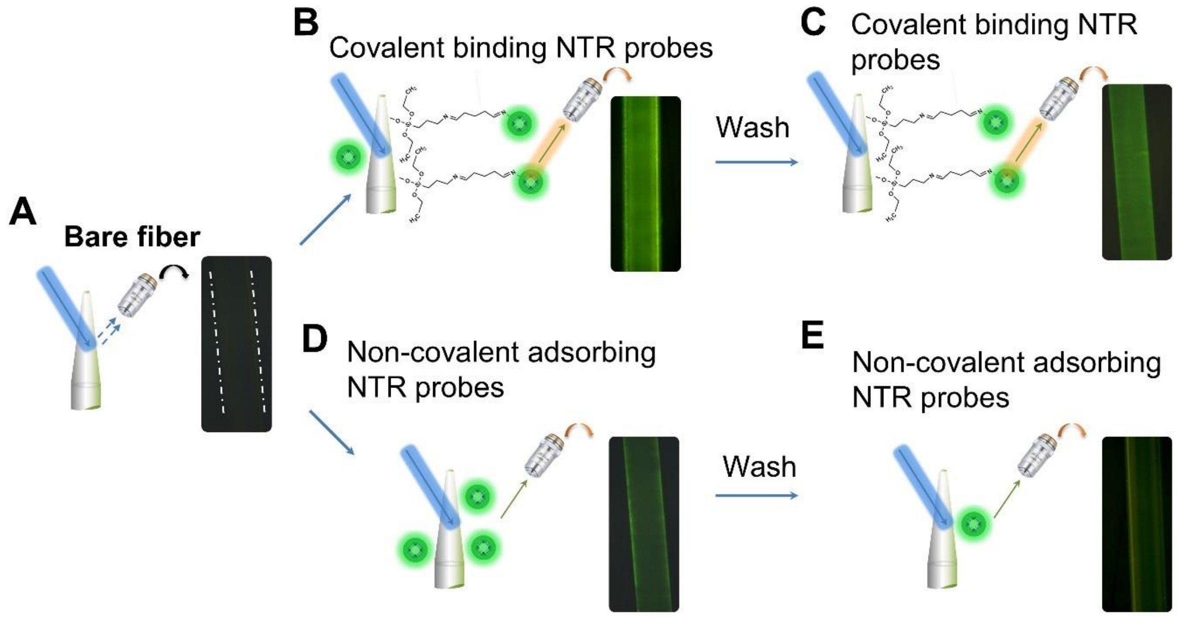

Figure S6. Characterization of the sensing probe under fluorescent microscope. A) the original fiber (Dark). B) Fiber with covalent binding of NTR probes (Bright green). C) Fiber with covalent binding of NTR probes after washing (Moderate green). D) Fiber with non-covalent adsorbing of NTR probes after rinsing in the NTR probes solution (Weak green). E) Fiber with non-covalent adsorbing of NTR probes after washing (Dim green).

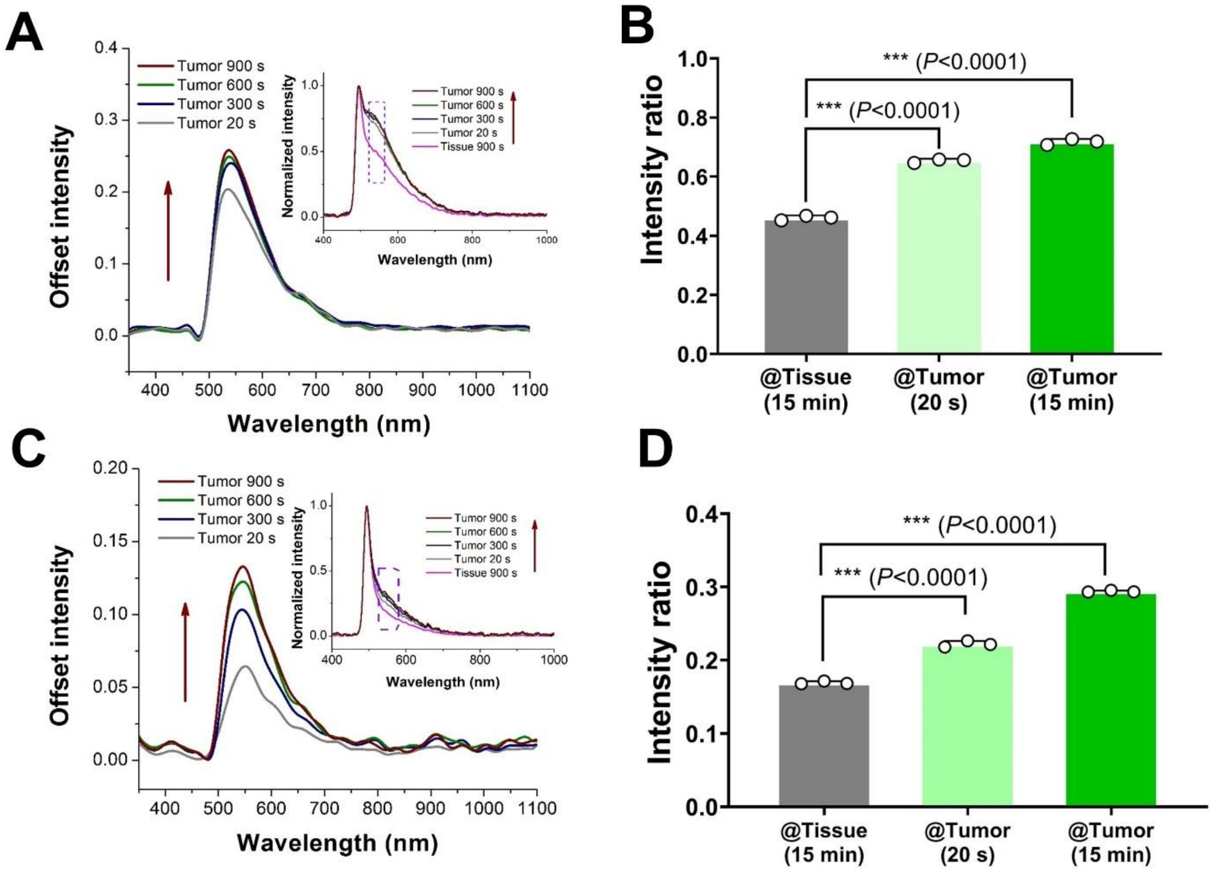

Figure S7. Repeated *in vivo* test using another fiber sensor fabricated by the same functional procedures: A) fluorescent spectra of the normal tissue spot with duration of 15 min and the tumor along with time. B) The comparison of the fluorescent signals from between the normal tissue test (at 15 min) and tumor test (20 s and 15 min); the recycled the fiber sensor from the one described in the main text (Figure 2D). C) fluorescent spectra of the normal tissue spot with duration of 15 min and the tumor along with time. D) The comparison of the fluorescent signals from between the normal tissue test (at 15 min) and tumor test (20 s and 15 min). ***$P<0.0001$

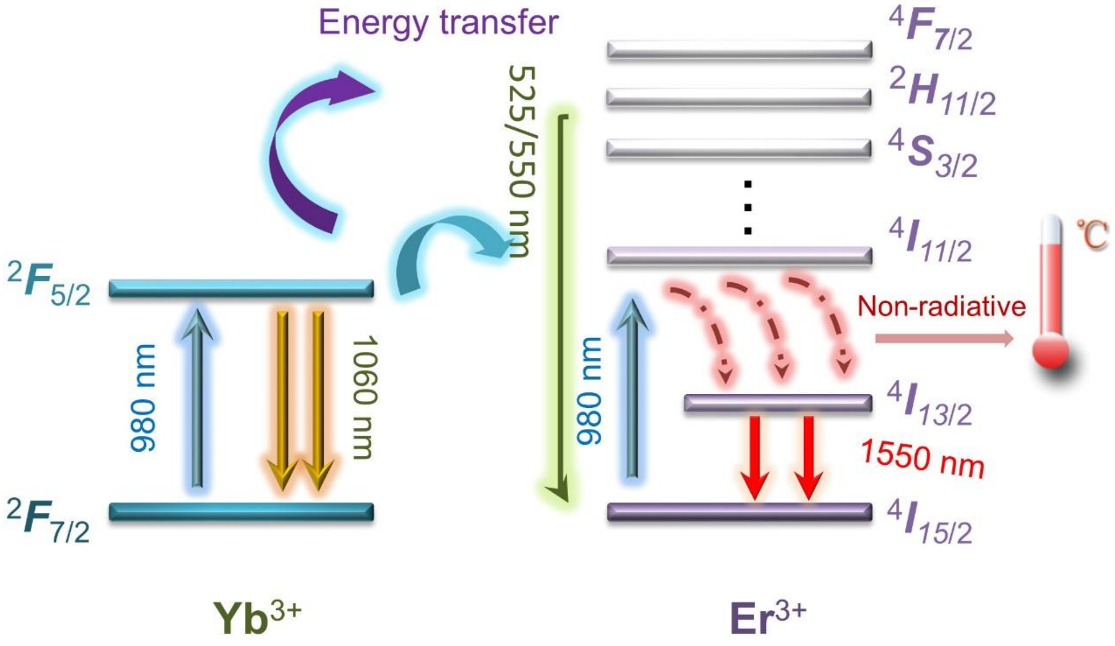

Figure S8. Scheme of the energy level and transition of the Yb and Er dopants.

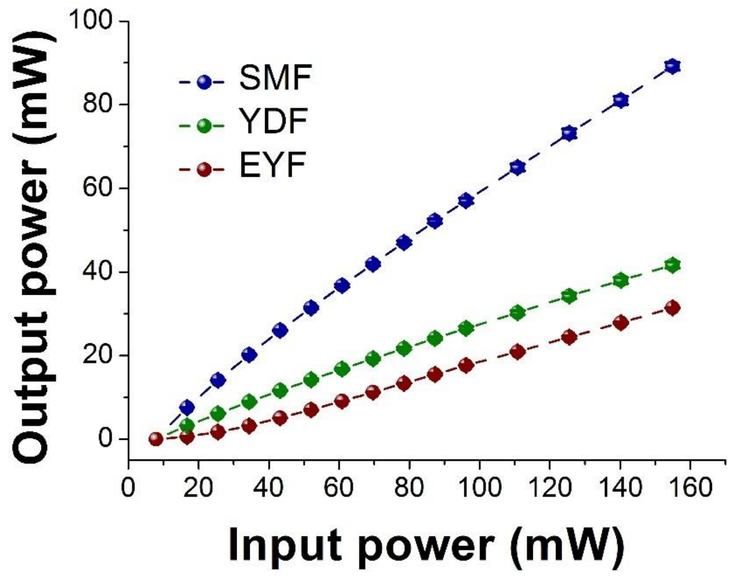

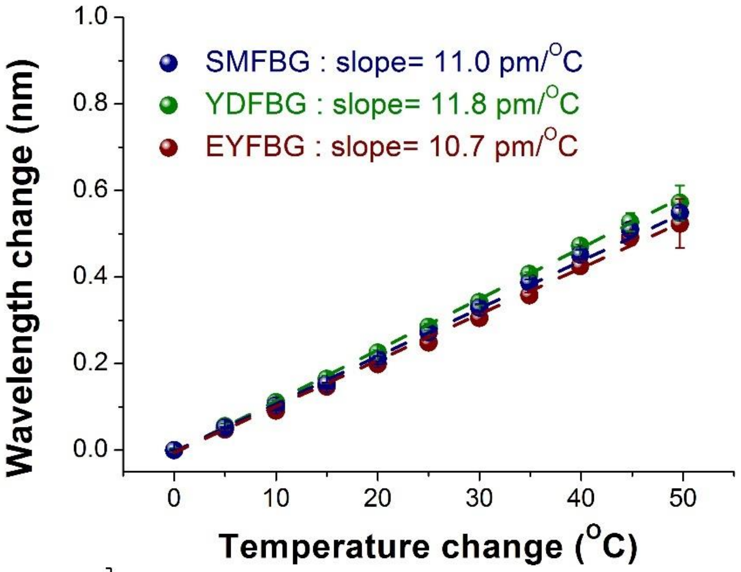

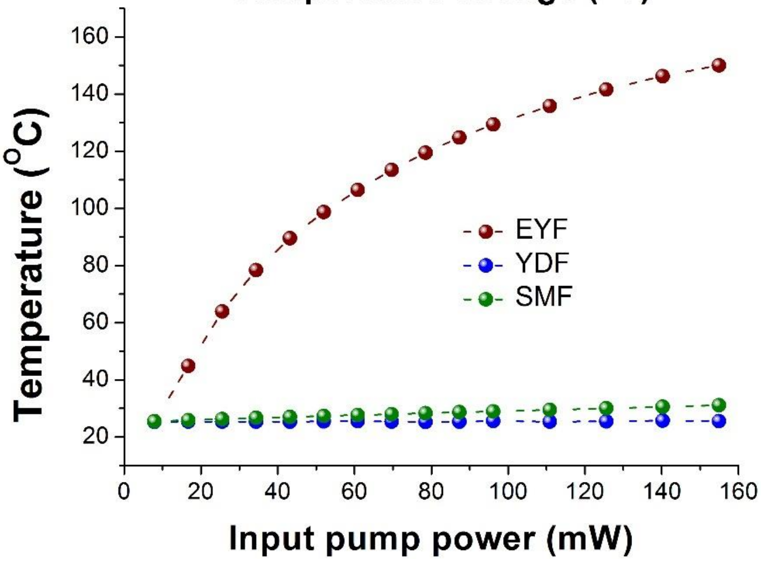

Figure S9. Characterization of the fibers with build-in FBGs. Top: The pump laser absorption using different fibers. Middle: Temperature sensitivity of the FBG inscribed into those fibers. Bottom: Photon-induced temperature changes in the air using those kinds of fibers as a function of the pump power. SMF, the telecom single mode fiber; YDF, a kind of Yb mono-doped active fiber; EYF, a kind of Er and Yb codoped fiber.

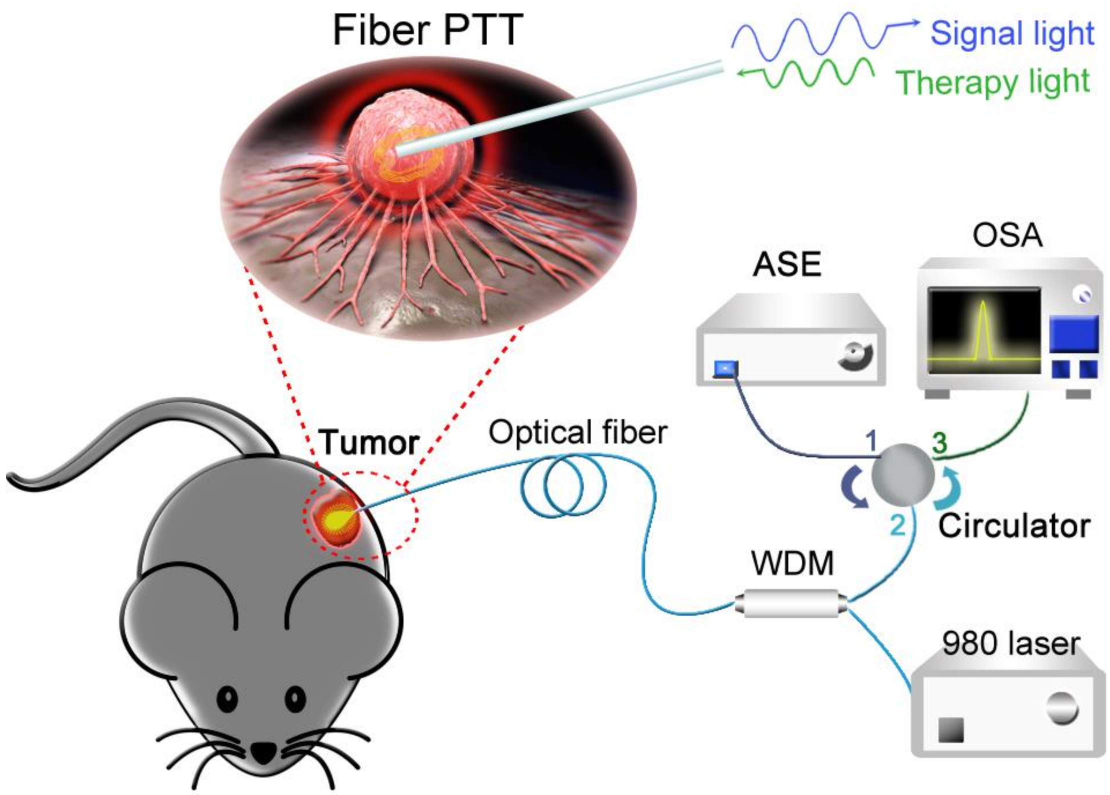

Figure S10. Diagram of the fiber PTT setup. ASE: amplifed spontenous emission; OSA: optical spectral analyzer; WDM: wavelength divider and multiplexer.

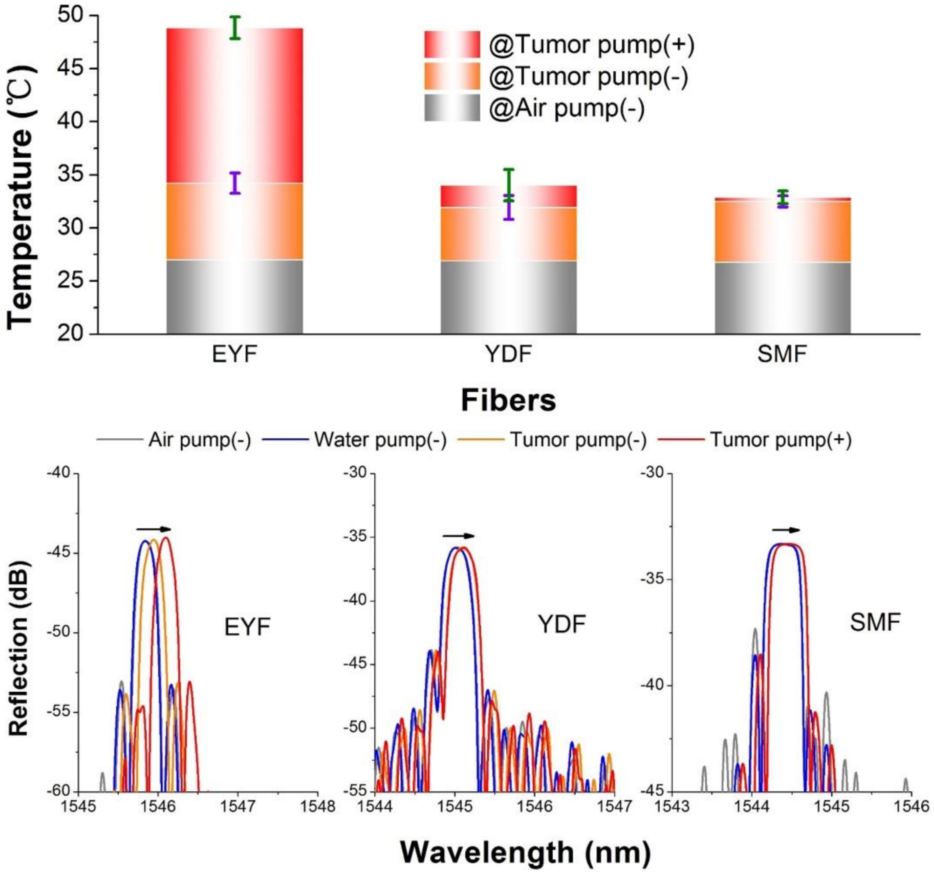

Figure S11. Temperature changes monitored by the built-in FBGs regarding the scenario shift from air to tumor and then in tumor photo-heating.

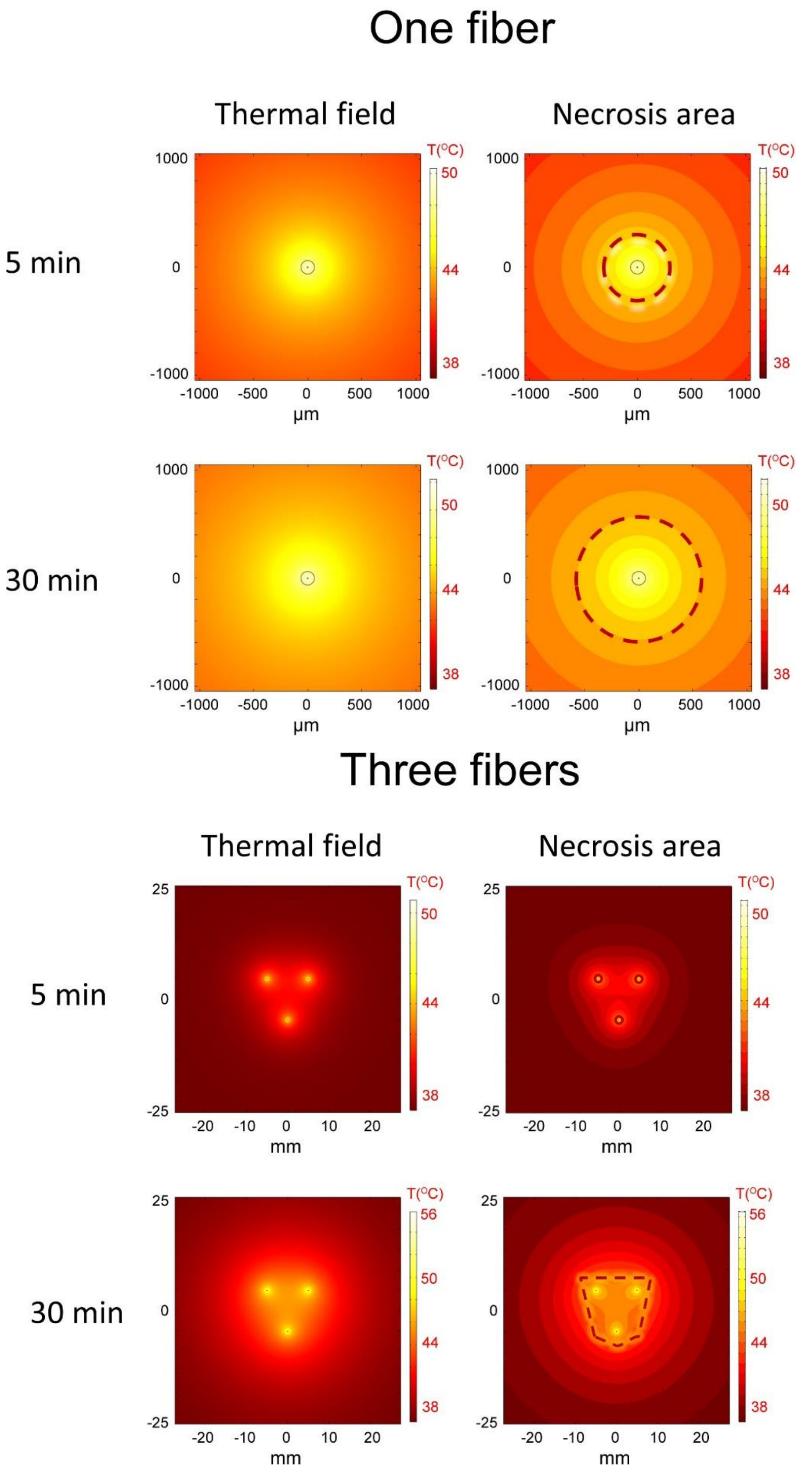

Figure S12. Simulation of the PTT effect contrast using one and three active fibers.

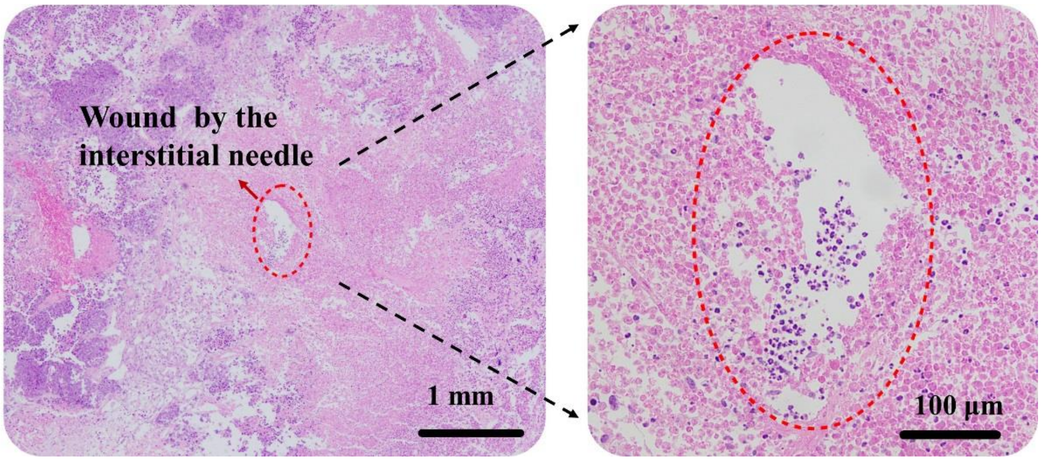

Figure S13. Histological analysis of the therapeutic effect area around the PTT fiber in the PANC-1 tumor section after the treatment. The red-dash circle indicated the wound as a result of the needle invasion.

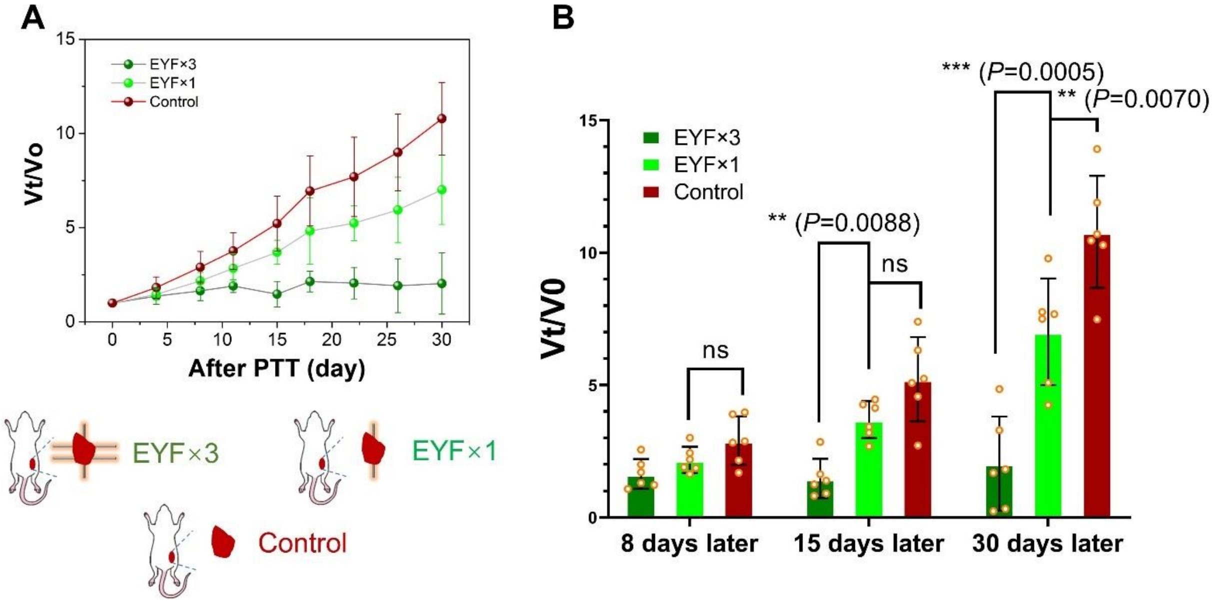

Figure S14. Comparison of the therapy outcome between the quantity of the PPT fiber usage. A) Although the single fiber PTT showed a certain suppression to the tumor volume, the residual tumor still grew after treatment. B) Comparing the tumor volume ratio at different time after treatment. The one fiber treatment showed no significance to the control group until the 30th day, denoting that the interior of the tumor was locally killed. By contrast, the three-fiber PTT exhibited significantly difference to the one fiber PTT at the 15th day. $^{**}P<0.01$, $^{***}P<0.001$.

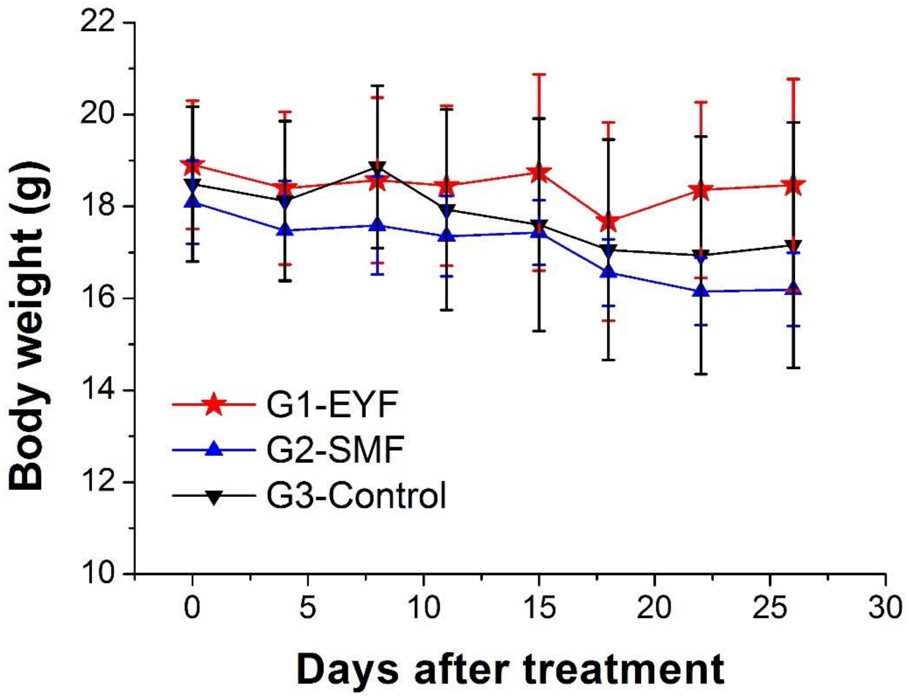

Figure S15. The changes of body weight of the mice after the therapy treatment in different groups. The body weights of mice showed negligible changes during the therapy cycle.

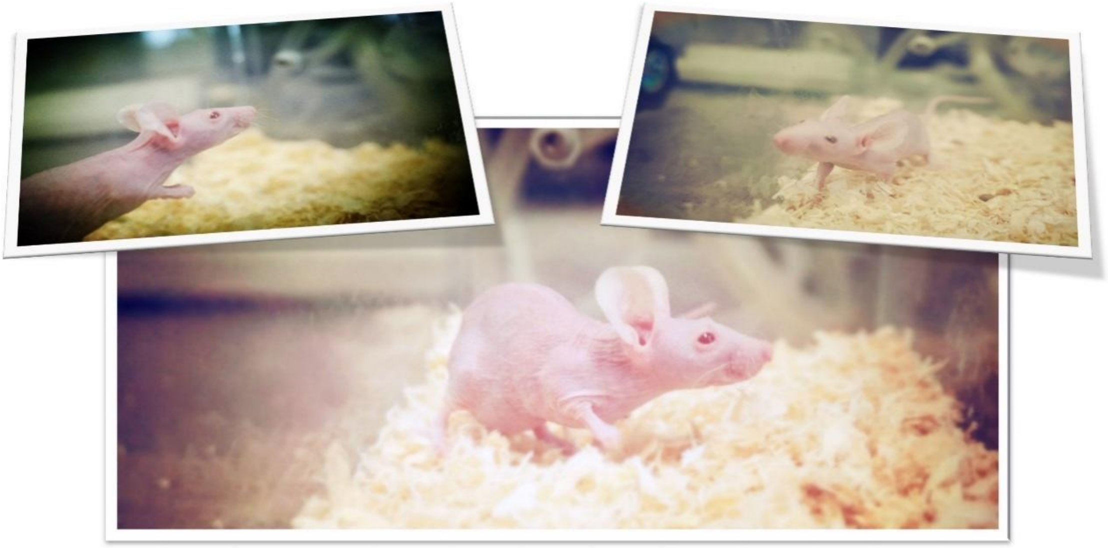

Figure S16. Photographs of the first cured mouse- “Aceso”-in our work. “Aceso” had survived over 320 days (Nov. 27th, 2019 to Oct. 20th, 2020) after the PTT without any tumor recurrence.

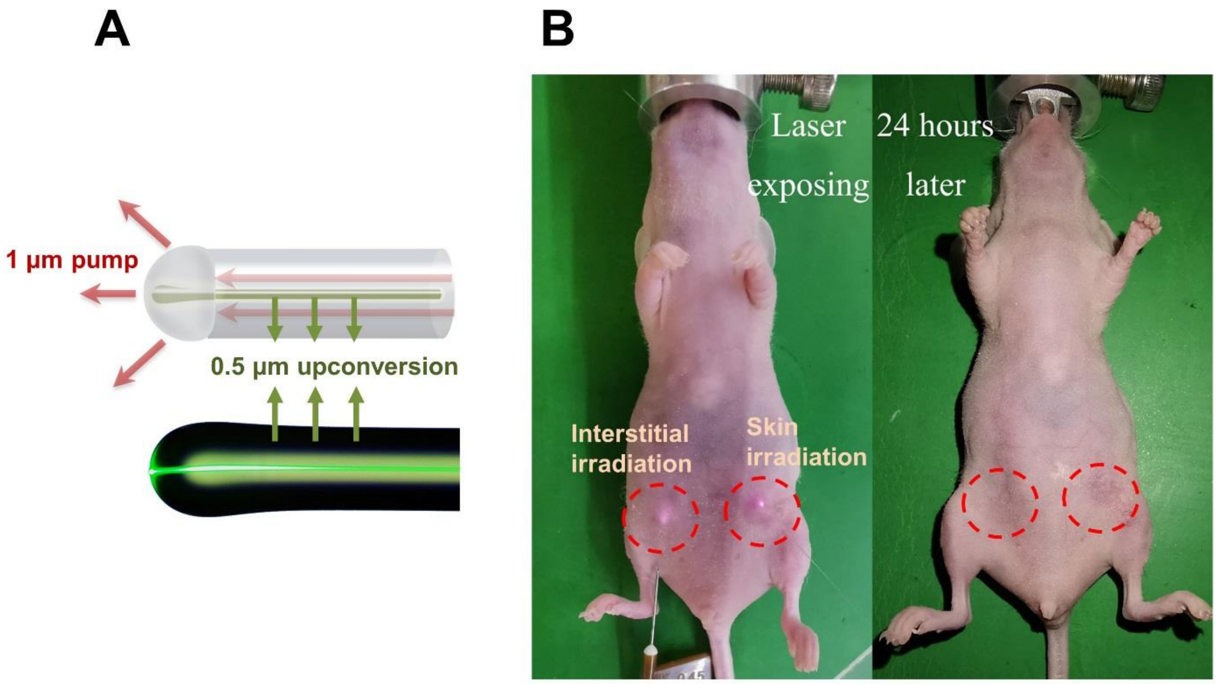

Figure S17. Safety of the residual scattered pump laser for the future fiber endoscopic treatment. A) Fiber end-face tailoring for scattering the residual pump laser output and the lateral green emission of the fiber-end relying on the upconversion of the rare earth dopants. B) The scattered residual pump laser reduced the power density to enhance safety using fiber PTT. Left flank: lasing within the tissue; right flank: lasing on the skin directly.

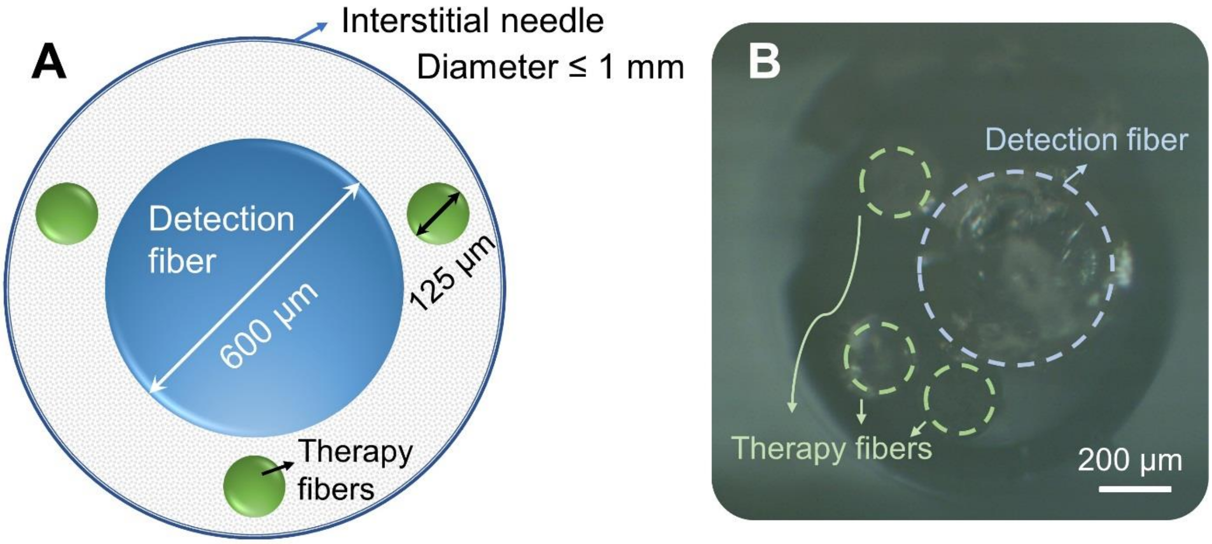

Figure S18. A) Diagram and B) SEM image, of the cross-section of the integrated fiber theranostic needle. The fluorescent fiber probe with the diameter of 150-600 μm and rare-earth fibers with the diameter of 125 μm were arranged side-by-side into a syringe needle with a whole diameter less than 1 mm.

Supplementary video

1. The demonstration of the process of the fiber PTT manipulation and temperature governing.

2. The proof-of-the-concept of the fiber-optic theranostic needles integrated into one syringe needle with a diameter of 1mm.

References

[34] M. Hu, C. Yang, Y. Luo, F. Chen, F. Yang, S. Yang, H. Chen, Z. Cheng, K. Li, Y. Xie, Journal of Materials Chemistry B **2018**, 6, 2413.
[35] K. Kiyose, K. Hanaoka, D. Oushiki, T. Nakamura, M. Kajimura, M. Suematsu, H. Nishimatsu, T. Yamane, T. Terai, Y. Hirata, T. Nagano, Journal of the American Chemical Society **2010**, 132, 15846.
[36] Z. Li, X. Li, X. Gao, Y. Zhang, W. Shi, H. Ma, Analytical Chemistry **2013**, 85, 3926.
[37] S. Kizaka-Kondoh, H. Konse-Nagasawa, Cancer Science **2009**, 100, 1366.
[38] W. R. Wilson, M. P. Hay, Nature Reviews Cancer **2011**, 11, 393.
[39] L. Luo, F. Song, X. Yu, W. Wang, C. Ming, L. Han, Y. Yu, H. Wu, J. Tian, Journal of Applied Physics **2010**, 107, 033110.
[40] L. Zenteno, Journal of Lightwave Technology **1993**, 11, 1435.
[41] Y. Ran, Z. Xu, F. Feng, P. Xiao, Y. Liang, L. Jin, B.-O. Guan, Opt. Lett. **2018**, 43, 2787.
[42] Y. Zhang, Y. Yin, J. Cai, X. Long, Z. Xu, Y. Ran, B. O. Guan, IEEE Photonics Journal **2021**, 13, 6800309.
[43] E. Desurvire, *Erbium-doped fiber amplifiers: principles and applications*, John Wiley and sons, New York **1994**.
[44] D. Ming, P. K. Cheo, IEEE Photonics Technology Letters **1997**, 9, 324.
[45] E. Downing, L. Hesselink, J. Ralston, R. Macfarlane, Science **1996**, 273, 1185.
[46] G. Yi, B. Sun, F. Yang, D. Chen, Y. Zhou, J. Cheng, Chemistry of Materials **2002**, 14, 2910.
[47] R. Kapoor, C. S. Friend, A. Biswas, P. N. Prasad, Opt. Lett. **2000**, 25, 338.
[48] M. Pollnau, D. R. Gamelin, S. R. Lüthi, H. U. Güdel, M. P. Hehlen, Physical Review B **2000**, 61, 3337.
[49] M.-F. Joubert, Optical Materials **1999**, 11, 181.
[50] H. U. Güdel, M. Pollnau, Journal of Alloys and Compounds **2000**, 303-304, 307.
[51] B.-S. Moon, T. K. Lee, W. C. Jeon, S. K. Kwak, Y.-J. Kim, D.-H. Kim, Nature Communications **2021**, 12, 4437.
[52] L. Qi, L. Jin, Y. Liang, L. Cheng, B. Guan, IEEE Photonics Technology Letters **2014**, 26, 1188.
[53] E. M. Knavel, C. L. Brace, Techniques in Vascular and Interventional Radiology **2013**, 16, 192.
[54] L. Meng, Y. Wu, T. Yi, Chemical Communications **2014**, 50, 4843.

References

[1] R. Ackroyd, C. Kelty, N. Brown, M. Reed, Photochemistry and Photobiology **2001**, 74, 656.
[2] Y. Liu, P. Bhattarai, Z. Dai, X. Chen, Chemical Society Reviews **2019**, 48, 2053.
[3] H. Shi, P. J. Sadler, British Journal of Cancer **2020**, 123, 871.
[4] X. Bao, Y. Yuan, J. Chen, B. Zhang, D. Li, D. Zhou, P. Jing, G. Xu, Y. Wang, K. Holá, D. Shen, C. Wu, L. Song, C. Liu, R. Zbořil, S. Qu, Light: Science & Applications **2018**, 7, 91.
[5] S. Li, W. Su, H. Wu, T. Yuan, C. Yuan, J. Liu, G. Deng, X. Gao, Z. Chen, Y. Bao, F. Yuan, S. Zhou, H. Tan, Y. Li, X. Li, L. Fan, J. Zhu, A. T. Chen, F. Liu, Y. Zhou, M. Li, X. Zhai, J. Zhou, Nature Biomedical Engineering **2020**, 4, 704.
[6] Z. Wang, Z. Li, Z. Sun, S. Wang, Z. Ali, S. Zhu, S. Liu, Q. Ren, F. Sheng, B. Wang, Y. Hou, Science Advances **2020**, 6, eabc8733.
[7] H. Xiang, L. Zhao, L. Yu, H. Chen, C. Wei, Y. Chen, Y. Zhao, Nature Communications **2021**, 12, 218.
[8] R. Weissleder, Nature Biotechnology **2001**, 19, 316.
[9] L. Wu, X. Qu, Chemical Society Reviews **2015**, 44, 2963.
[10] S. He, J. Li, Y. Lyu, J. Huang, K. Pu, Journal of the American Chemical Society **2020**, 142, 7075.
[11] F. J. Voskuil, P. J. Steinkamp, T. Zhao, B. van der Vegt, M. Koller, J. J. Doff, Y. Jayalakshmi, J. P. Hartung, J. Gao, B. D. Sumer, M. J. H. Witjes, G. M. van Dam, Y. Albaroodi, L. B. Been, F. Dijkstra, B. van Etten, Q. Feng, R. J. van Ginkel, K. Hall, K. Havenga, J. W. Haveman, P. H. J. Hemmer, L. Jansen, S. J. de Jongh, G. Kats-Ugurlu, W. Kelder, S. Kruijff, I. Kruithof, E. van Loo, J. L. N. Roodenburg, N. Shenoy, K. P. Schepman, S. A. H. J. de Visscher, S. s. g. the, Nature Communications **2020**, 11, 3257.
[12] P. Dharmalingam, K. Venkatakrishnan, B. Tan, Nano Letters **2020**, 20, 1054.
[13] N. M. Ralbovsky, I. K. Lednev, Chemical Society Reviews **2020**, 49, 7428.
[14] D. Lin, C.-L. Hsieh, K.-C. Hsu, P.-H. Liao, S. Qiu, T. Gong, K.-T. Yong, S. Feng, K. V. Kong, Nature Communications **2021**, 12, 3430.
[15] X. Li, J. F. Lovell, J. Yoon, X. Chen, Nature Reviews Clinical Oncology **2020**, 17, 657.
[16] Y. Jiang, J. Huang, C. Xu, K. Pu, Nature Communications **2021**, 12, 742.
[17] J. Karges, S. Kuang, F. Maschietto, O. Blacque, I. Ciofini, H. Chao, G. Gasser, Nature Communications **2020**, 11, 3262.
[18] F. Peng, M. I. Setyawati, J. K. Tee, X. Ding, J. Wang, M. E. Nga, H. K. Ho, D. T. Leong, Nature Nanotechnology **2019**, 14, 279.
[19] Y. Zhao, X.-g. Hu, S. Hu, Y. Peng, Biosensors and Bioelectronics **2020**, 166, 112447.
[20] J. Zou, W. Li, N. Meng, J. Jiang, C. Wu, T. C. Lei, R. Sroka, Z. Huang, Photodiagnosis and Photodynamic Therapy **2020**, 31, 101924.
[21] A. L. Chin, S. Jiang, E. Jang, L. Niu, L. Li, X. Jia, R. Tong, Nature Communications **2021**, 12, 5138.
[22] R. Gassino, Y. Liu, M. Konstantaki, A. Vallan, S. Pissadakis, G. Perrone, Journal of Lightwave Technology **2017**, 35, 3447.
[23] H. Li, Y. Huang, G. Hou, A. Xiao, P. Chen, H. Liang, Y. Huang, X. Zhao, L. Liang, X. Feng, B.-O. Guan, Science Advances **2019**, 5, eaax4659.
[24] Y. Li, Y. Sun, J. Li, Q. Su, W. Yuan, Y. Dai, C. Han, Q. Wang, W. Feng, F. Li, Journal of the American Chemical Society **2015**, 137, 6407.
[25] S. Banerjee, E. B. Veale, C. M. Phelan, S. A. Murphy, G. M. Tocci, L. J. Gillespie, D. O. Frimannsson, J. M. Kelly, T. Gunnlaugsson, Chemical Society Reviews **2013**, 42, 1601.
[26] X. Yang, C. Gong, C. Zhang, Y. Wang, G.-F. Yan, L. Wei, Y.-C. Chen, Y.-J. Rao, Y. Gong, Laser & Photonics Reviews **2022**, 16, 2100171.

[27] Y. Yu, F. Fu, L. Shang, Y. Cheng, Z. Gu, Y. Zhao, Advanced Materials **2017**, 29, 1605765.
[28] Y. Yu, L. Shang, J. Guo, J. Wang, Y. Zhao, Nature Protocols **2018**, 13, 2557.
[29] J. Xiao, T. Zhou, N. Yao, S. Ma, C. Pan, P. Wang, H. Fu, H. Liu, J. Pan, L. Yu, S. Wang, W. Yang, L. Tong, L. Zhang, Nature Communications **2022**, 13, 363.
[30] D. Nambiar, V. Prajapati, R. Agarwal, R. P. Singh, Cancer Lett **2013**, 334, 109.
[31] S. Frentzas, E. Simoneau, V. L. Bridgeman, P. B. Vermeulen, S. Foo, E. Kostaras, M. R. Nathan, A. Wotherspoon, Z.-h. Gao, Y. Shi, G. Van den Eynden, F. Daley, C. Peckitt, X. Tan, A. Salman, A. Lazaris, P. Gazinska, T. J. Berg, Z. Eltahir, L. Ritsma, J. van Rheenen, A. Khashper, G. Brown, H. Nyström, M. Sund, S. Van Laere, E. Loyer, L. Dirix, D. Cunningham, P. Metrakos, A. R. Reynolds, Nature Medicine **2016**, 22, 1294.
[32] L. Yan, C. H. Lee, Journal of Applied Physics **1994**, 75, 1286.
[33] M. Innocenzi, H. Yura, C. Fincher, R. Fields, Applied Physics Letters **1990**, 56, 1831.
[34] M. Hu, C. Yang, Y. Luo, F. Chen, F. Yang, S. Yang, H. Chen, Z. Cheng, K. Li, Y. Xie, Journal of Materials Chemistry B **2018**, 6, 2413.
[35] K. Kiyose, K. Hanaoka, D. Oushiki, T. Nakamura, M. Kajimura, M. Suematsu, H. Nishimatsu, T. Yamane, T. Terai, Y. Hirata, T. Nagano, Journal of the American Chemical Society **2010**, 132, 15846.
[36] Z. Li, X. Li, X. Gao, Y. Zhang, W. Shi, H. Ma, Analytical Chemistry **2013**, 85, 3926.
[37] S. Kizaka-Kondoh, H. Konse-Nagasawa, Cancer Science **2009**, 100, 1366.
[38] W. R. Wilson, M. P. Hay, Nature Reviews Cancer **2011**, 11, 393.
[39] L. Luo, F. Song, X. Yu, W. Wang, C. Ming, L. Han, Y. Yu, H. Wu, J. Tian, Journal of Applied Physics **2010**, 107, 033110.
[40] L. Zenteno, Journal of Lightwave Technology **1993**, 11, 1435.
[41] Y. Ran, Z. Xu, F. Feng, P. Xiao, Y. Liang, L. Jin, B.-O. Guan, Opt. Lett. **2018**, 43, 2787.
[42] Y. Zhang, Y. Yin, J. Cai, X. Long, Z. Xu, Y. Ran, B. O. Guan, IEEE Photonics Journal **2021**, 13, 6800309.
[43] E. Desurvire, *Erbium-doped fiber amplifiers: principles and applications*, John Wiley and sons, New York **1994**.
[44] D. Ming, P. K. Cheo, IEEE Photonics Technology Letters **1997**, 9, 324.
[45] E. Downing, L. Hesselink, J. Ralston, R. Macfarlane, Science **1996**, 273, 1185.
[46] G. Yi, B. Sun, F. Yang, D. Chen, Y. Zhou, J. Cheng, Chemistry of Materials **2002**, 14, 2910.
[47] R. Kapoor, C. S. Friend, A. Biswas, P. N. Prasad, Opt. Lett. **2000**, 25, 338.
[48] M. Pollnau, D. R. Gamelin, S. R. Lüthi, H. U. Güdel, M. P. Hehlen, Physical Review B **2000**, 61, 3337.
[49] M.-F. Joubert, Optical Materials **1999**, 11, 181.
[50] H. U. Güdel, M. Pollnau, Journal of Alloys and Compounds **2000**, 303-304, 307.
[51] B.-S. Moon, T. K. Lee, W. C. Jeon, S. K. Kwak, Y.-J. Kim, D.-H. Kim, Nature Communications **2021**, 12, 4437.
[52] L. Qi, L. Jin, Y. Liang, L. Cheng, B. Guan, IEEE Photonics Technology Letters **2014**, 26, 1188.
[53] E. M. Knavel, C. L. Brace, Techniques in Vascular and Interventional Radiology **2013**, 16, 192.
[54] L. Meng, Y. Wu, T. Yi, Chemical Communications **2014**, 50, 4843.
